# Finite Time Blow-Up for the Hypodissipative Navier Stokes Equations with a Force in $L^1_t C_x^{1,\epsilon }\cap L^{\infty }_{t} L^{2}_{x}$

**DOI:** 10.1007/s00205-026-02198-0

**Published:** 2026-05-11

**Authors:** Diego Córdoba, Luis Martínez-Zoroa, Fan Zheng

**Affiliations:** 1 Instituto de Ciencias Matemáticas CSIC-UAM-UCM-UC3M, Madrid, Spain; 2 CUNEF Universidad, Madrid, Spain

## Abstract

In this work we establish the formation of singularities of classical solutions with finite energy of the forced fractional Navier Stokes equations where the dissipative term is given by $|\nabla |^{\alpha }$ for any $\alpha \in [0, \alpha _0)$ ($\alpha _0 = \frac{22-8\sqrt{7}}{9} > 0$). We construct solutions in $\mathbb {R}^3\times [0,T]$ with a finite $T>0$ and with an external forcing which is in $L^1_t([0, T]) C_x^{1,\epsilon }\cap L^{\infty }_{t} L^{2}_{x}$, such that for each time $t \in [0, T)$, the velocity *u* is in the space $C^\infty \cap L^2$ and such that as the time *t* approaches the blow-up moment *T*, the integral $\int _0^t |\nabla u| \text {d}s$ tends to infinity. Since this result lays in a well-posedness class, the blow-up is generated by the dynamics of the equation and not by the force itself. This is the first blow-up result for hypodissipative Navier–Stokes in a well-posedness class.

## Introduction

We consider the initial value problem for the fractional Navier–Stokes equations in $\mathbb {R}^{3}$,

1 $$\begin{aligned} \begin{aligned} u_t+u\cdot \nabla u + \nabla P + \nu |\nabla |^{\alpha } u&= f, \quad (x,t) \in \mathbb {R}^{3} \times \mathbb {R}_{+}, \\ \quad \quad \quad \nabla \cdot u&= 0 \end{aligned} \end{aligned}$$

with initial data $u_0= u(x,0)$, where

$$\begin{aligned} u&=(u_1(x,t), u_2(x,t), u_3(x,t))\text { is the velocity field},\\ P&=P(x,t)\text { is the pressure function},\\ \nu&>0\text { is the viscosity coefficient},\\ f&=(f_1(x,t), f_2(x,t), f_3(x,t))\text { is an external force}. \end{aligned}$$

We let $|\nabla |^{\alpha } g\equiv (-\Delta )^{\frac{\alpha }{2}} g$ be the Fourier multiplier $\widehat{|\nabla |^{\alpha } g}(\xi ) = |\xi |^{\alpha }\widehat{g} (\xi )$.

When $\alpha =2$, the equations align with the well-recognized classical Navier–Stokes equations. If $0<\alpha <2$, these equations are called hypodissipative Navier–Stokes equations, indicating their reduced kinetic energy dissipation compared to the complete Laplacian $\Delta$. From a physical perspective, this model characterizes fluids that exhibit internal friction as described in [31] and has also been derived using a stochastic Lagrangian particle methodology according to [43]. On the other hand, if $\alpha >2$, the equations are considered hyperdissipative. When $\nu =0$ we have the incompressible Euler equations.

We can deduce their corresponding energy equality

$$\begin{aligned}&\frac{1}{2}\int _{\mathbb {R}^{3}} |u(x,t)|^2 \text {d}x + \nu \int _0^t\int _{\mathbb {R}^{3}} ||\nabla |^{\alpha /2} u(x,s)|^2 \text {d}x\text {d}s \\&\quad = \frac{1}{2}\int _{\mathbb {R}^{3}} |u(x,0)|^2 \text {d}x +\int _0^t\int _{\mathbb {R}^{3}} u(x,s)f(x,s) \text {d}x\text {d}s \end{aligned}$$

for smooth solutions by multiplying the initial, equation by u and performing integration by parts. Also, by taking the curl of the equation we get its vorticity formulation

2 $$\begin{aligned} \omega _t+u\cdot \nabla \omega + \nu |\nabla |^{\alpha } \omega = \nabla \times f + \omega \cdot \nabla {u}, \end{aligned}$$

where *u* can now be recovered from $\omega$ using the Biot–Savart law

$$ u(x) = \omega * \mathcal {K}(x) = \frac{1}{4\pi }\int _{\mathbb {R}^3} \frac{\omega (y) \times (x - y)}{|x - y|^3}\text {d}y, $$

where $\mathcal {K}$ is the Biot–Savart kernel.

The term $\omega \cdot \nabla u$ on the right-hand side means that in addition to being transported by the velocity, the vorticity itself will be stretched by the deformation caused by the flow; hence its name “the vorticity stretching term". This term is peculiar to three dimensions; in two dimensions the vorticity always points perpendicularly to the flow, so the vorticity stretching term vanishes. This explains the stark contrast in the global regularity theory in two and three dimensions: in two dimensions global well-posedness is well known [23, 42], while in three dimensions the corresponding problem is the famous Millennium Prize Problems [22]. If the initial velocity $u_0$ is smooth and the external force *f* is uniformly smooth, does a smooth solution to these equations exist for all time, or is there a finite time singularity? This question is a cornerstone of mathematical fluid dynamics and remains unsolved, stimulating extensive research to shed light on the potential existence or non-existence of singularities.

Following the groundbreaking work by J. Leray [29], a wealth of extensive literature has been developed on the existence theory for solutions of the Navier–Stokes equations. A significant advancement in regularity theory is the renowned partial-regularity theory developed by Caffarelli–Kohn–Nirenberg [3], who proved that the 1-dimensional parabolic Hausdorff measure of the singular set (the points (*x*, *t*) where *u* is unbounded in any neighborhood of (*x*, *t*) is zero) for every suitable weak solution. More recently, this theory has been expanded in [28, 37, 38] to the hypodissipative range $\frac{3}{2}< \alpha < 2$ by proving that the $(5 - 2\alpha )$-dimensional Hausdorff measure of the singular set is zero for every suitable weak solution. In the scenario of hyperdissipation, where $2 < \alpha \le \frac{5}{2}$, a parallel achievement has been recently shown in [11] (see also [25, 34]).

The balance of energy in the hyperdissipative Navier–Stokes equation reaches a critical scale at $\alpha = \frac{5}{2}$. For this equation, global regularity is maintained for all solutions when $\alpha \ge \frac{5}{2}$ (see the work of Lions [30]), extending logarithmically into the range beyond the critical threshold (refer to [1, 21, 39]). Additionally, as demonstrated in [10], given any specific initial condition, $u_0$, belonging to the space $H^{\delta }$ with $\delta > 0$, such initial condition $u_0$ ensures the emergence of a globally smooth solution whenever $\alpha$ exceeds $\frac{5}{2} - \epsilon$, with $\epsilon$ being a small positive number determined solely by the $H^{\delta }$ magnitude of the initial condition.

Three significant results concerning the propagation of regularity for Navier–Stokes equations are the Prodi-Serrin criteria on the integrability of the velocity (see also [16] and reference [35] for details and reviews on the criteria), the criterion on the direction field of the vorticity by Constantin–Fefferman in [12] and the Seregin-Sverak criteria in [36] on the lower bound of the pressure. These have been extensively extended in various studies to the fractional Navier–Stokes equation (see [2, 10, 27, 33, 41, 43] for further details and references therein). Conversely, there exist models of the fractional and classical Navier–Stokes equations that develop finite time singularities, in particular, see [40] by Tao for a modified averaged Navier–Stokes system and for a hypodissipative dyadic model see both [26] by Katz-Pavlovic and [8] by Cheskidov (see also [9, 32]). Very recently a potential candidate for a finite time singularity for the classical Navier–Stokes equations has been presented in Hou [24].

The goal of this paper is to construct classical solutions in the well-posed regime of the hypodissipative Navier–Stokes equations that loses regularity in finite time. The main theorem is

### Theorem 1

There is $\alpha _0 = \frac{22-8\sqrt{7}}{9} > 0$ such that for any $\alpha \in [0,\alpha _0)$, there exists a solution of the forced 3D incompressible fractional Navier–Stokes equations (1) in $\mathbb {R}^3\times [0,1]$ with $\nu >0$^1^ and an external forcing which is in $L^1_t([0, 1]) C_x^{1,\epsilon }\cap L^{\infty }_{t}L^{2}_{x}$ for some $\epsilon >0$, such that the velocity $u \in L_{t,x}^\infty \cap L_t^\infty L_x^2$, and that on the time interval $0 \le t < 1$, the velocity *u* is smooth in *x* and satisfies

$$\begin{aligned} \lim _{t\rightarrow 1}\int _0^t |\nabla u (x,s)|_{L^{\infty }} \text {d}s = \infty . \end{aligned}$$

### Remark 1

In particular, at the time of blow-up $t=1$, the solution *u* is no longer $C^1$. In fact it is only $C^{1-\sqrt{2\alpha /7}}$.

### Some Comments About the Result and Hypodissipative Navier–Stokes

Note that, using standard methods, there is local existence for hypodissipative Navier–Stokes in $L_t^\infty C_x^{1,\epsilon }$ if the force is in $L_t^1C_x^{1,\epsilon }$ (see for example [15], where Proposition 3.5 proves local existence in $C^{1,\alpha }$, and note that proposition 3.3 in the same paper allows extending the result to the forced case in a straightforward way), so our blow up cannot be attributed to the force. In particular, for hypodissipative Navier–Stokes, there is a lower bound for the time of existence depending only on $\Vert u_{0}\Vert _{C^{1,\epsilon }}$ and $\Vert f\Vert _{L^{1}_{t}C^{1,\epsilon }}$, and a blow-up happens at time *T* if and only if

$$\begin{aligned} \text {lim}_{t\rightarrow T}\int _{0}^{t}\Vert u\Vert _{C^{1}}=\infty . \end{aligned}$$

Therefore, this result can be understood as a blow-up result in spaces of subcritical regularity, as far as we know, it is the only such result that has been proved so far for hypodissipative Navier–Stokes, or any incompressbile fluid models.

It is also worth noting that, before the time of blow-up, both our forcing *f* and our solution *u* remain smooth.

Although this construction uses similar techniques as [13], the addition of the fractional diffusion creates significant new challenges, and in fact the construction from [13] does not allow for the addition of any fractional diffusion. This is due to several key differences that appear when we introduce diffusion:

- In the construction in [13], the solution is composed of an infinite number of vortex layers, i.e. $\omega (x,t)=\sum _{n=0}^{\infty }\omega _n$, and the frequencies of the different layers are growing super-exponentially. This big difference in the frequencies of the different pieces results in relatively weak growth in each layer, and in particular it would be overpowered by any amount of diffusion introduced in the equation.
- Even if one considers layers with more similar frequencies, the new term included in the equation $\nu |\nabla |^{\alpha }\omega$ is more singular than $\omega$ itself (which already becomes singular at the time of blow-up), so including this term directly in the forcing would give a forcing well below the well-posedness regime for fractional Navier–Stokes. This means the effects of the new term must be included in the evolution of our solution.
- It is not obvious how $\nu |\nabla |^{\alpha }\omega$ can be included in the ansatz, since this operator is highly non-local and hard to estimate for general functions. In order to deal with this, we obtain a suitable local approximation of the operator that are valid for the specific family of functions that we are considering.
- Once the term $\nu |\nabla |^{\alpha }\omega$ is included in the evolution of our solutions, since it will oppose the growth of our solution, we will need our solutions to become very big in $C^{1-\alpha }$. This is especially important in our construction, since (as we will see later), one of the terms we want to absorb with the force in our construction will be a term coming from each $\omega _{i}$ interacting with itself, and these terms become significantly worse as we consider lower regularity at the time of blow-up.

### Previous Results on Finite Time Singularities for 3D Incompressible Euler Equations

Recently different scenarios of blow-up has been constructed within local well-posed regime ($C^{1,\beta }$) in the inviscid case ($\nu =0$): the 3D incompressible Euler equations. More precisely, there are 4 known scenarios of blow-up with finite energy and no boundaries:

- Elgindi pioneered the first demonstration of finite-time singularities in Euler equations through his research [17]. He analyzed flows with axi-symmetric properties and no swirl, focusing on velocity profiles within the $C^{1,\beta }$ Hölder space, where $\beta$ is chosen to be a very small number, and which are $C^{\infty }$ outside of a zero measure set. His findings revealed the possibility of exact self-similar blow-up solutions that possess infinite energy and are not influenced by external forces. It’s important to highlight that by applying a consistent non-zero $C^{1,\beta }$ external force, one can also derive solutions that blow up yet have finite energy, as Elgindi mentions in Remark 1.4 of his paper [17]. In subsequent research [18], Elgindi, along with Ghoul and Masmoudi, expanded on these results, showing that such self-similar blow-up solutions could be localized. By examining the stability of these solutions, they confirmed the emergence of finite-time singularities in the Euler equations without any external forces ($f = 0$), specifically for solutions with finite energy in the $C^{1,\beta }$ framework. Recent progress has led to significant advancements and computer assisted proofs in the study of self-similar singularities in axi-symmetric flows when boundaries are present (refer to [5–7, 19]).
- In our recent work [14], we introduced a novel blowup mechanism in $\mathbb {R}^3$ that diverges from the typical self-similar profile. We have constructed solutions for the 3D unforced incompressible Euler equations over the domain $\mathbb {R}^3\times [-T,0]$, where the velocity profiles lie within the function space $C^{\infty }(\mathbb {R}^3 \setminus {0})\cap C^{1,\beta }\cap L^2$, where $0<\beta \ll \frac{1}{3}$, for time intervals $t\in (-T,0)$. These solutions are smooth everywhere except at a singular point and develop finite-time singularities at $t=0$ (see also [4], where a self-similar singularity smooth outside of a point is constructed). Remarkably, while these solutions are axi-symmetric without swirl, they do not rely on self-similar profiles. They are made up instead of a sequence of vorticity regions increasingly closer to the origin, creating a hyperbolic saddle at the center. This configuration induces motion and deformation, affecting the inner vortices. Thus, the Euler equations’ dynamics under this scheme can be approximated by a solvable infinite system of ordinary differential equations, offering a precise backward-in-time analysis of the dynamics leading to the blow-up, thereby enabling a detailed proof of this singular behavior.
- In the recent study [13], we introduce a blow-up mechanism for the forced 3D incompressible Euler equations, specifically targeting non-axisymmetric solutions. We develop solutions in $\mathbb {R}^3$ in which the velocity in $C^{3,\frac{1}{2}}\cap L^2$ over the time interval [0, *T*), where $T > 0$ is a finite value, and these solutions are subject to a continuous force in $C^{1,\frac{1}{2} -\epsilon }\cap L^2$. Notably, this approach leads to a blow-up: as time *t* nears the critical moment *T*, the integral $\int _0^t |\nabla u| \text {d}s$ diverges, indicating infinite growth, yet the solution maintains smoothness everywhere except at the origin. Our blow-up construction does not employ self-similar coordinates and successfully addresses solutions beyond the $C^{1,\frac{1}{3}+}$ regularity, which is critical for axi-symmetric solutions without swirl.
- Very recently, Elgindi and Pasqualotto consider the setting of axi-symmetric flows with swirl. They proved in [20] a self-similar profile blow-up for solutions with velocity in $C^{1, \beta }$ where the singularity happens away from the origin. The solutions considered actually live in $H^{2+\delta }$, and therefore they are above the critical regularity for axi-symmetric solutions in a stronger sense than the usual $C^{1,\beta }$ solutions considered in other results. The strategy requires a computer assisted proof in order to prove the invertibility of a certain linear operator.

### Details About the Proof

#### Strategy of the Proof

We use a similar strategy as the one considered in [13]. Namely, we consider solutions composed of an infinite number of compactly supported smooth vorticities

$$\begin{aligned} \sum _{n=0}^{\infty }\omega _{n}(x,t), \end{aligned}$$

which we call vortex layers, fulfilling that, if $n_{2}<n_{1}$, then $\omega _{n_{1}}$ is much more concentrated around the origin than $\omega _{n_{2}}$. This allows us to obtain a good approximation for the interaction between different layers by using, for example, Taylor series expansions. This allows us to approximate the interactions between outer velocity and inner vorticity as

3 $$\begin{aligned} \begin{aligned} \sum _{j=0}^{k-1} (\omega _{j}* \mathcal {K})\cdot \nabla \omega _{k}&\approx U_{k}\cdot \nabla \omega _{k},\\ \omega _{k}\cdot \nabla \sum _{j=0}^{k-1} \omega _{j} * \mathcal {K}&\approx \omega _{k}\cdot \nabla U_{k}, \end{aligned} \end{aligned}$$

where $U_{k}$ is a a useful approximation for $\sum _{j=0}^{k-1}u(\omega _{j})$. The interaction between inner velocity and outer vorticity is negligible, because the inner vorticity exhibits a larger cancellation that makes the velocity it generates very small far away from the inner layer:

4 $$\begin{aligned} \sum _{j=0}^{k-1}\omega _{j}\cdot \nabla (\omega _{k} * \mathcal {K})\text { and }(\omega _{k} * \mathcal {K})\cdot \nabla \sum _{j=0}^{k-1}\omega _{j}\approx 0. \end{aligned}$$

Furthermore, we will choose the $\omega _{k}$ so that the self-interactions cancel to first order, so that

5 $$\begin{aligned} (\omega _{k} * \mathcal {K})\cdot \nabla \omega _{k}\text { and }\omega _{k}\cdot \nabla (\omega _{k} * \mathcal {K})\approx 0. \end{aligned}$$

If we, for now, ignore the contribution of the fractional dissipation, the approximations (3), (4) and (5) transforms the Eq. (2) to

6 $$\begin{aligned} \partial _{t}\omega _{k}+U_{k}\cdot \nabla \omega _{k}=\omega \cdot \nabla U_{k}, \end{aligned}$$

which can be also written as

7 $$\begin{aligned} \partial _{t}\omega _{k}+\sum _{j=0}^k (\omega _{j} * \mathcal {K})\cdot \nabla \omega _{k}+(\omega _{k} * \mathcal {K})\cdot \nabla \sum _{j=0}^k \omega _{j} =\omega _{k}\cdot \nabla \sum _{j=0}^k (\omega _{j} * \mathcal {K})+\sum _{j=0}^k \omega _{j}\cdot \nabla (\omega _{k} * \mathcal {K})+F_{k}, \end{aligned}$$

where the force is compensating the errors we created in our approximations. Note that, if we add all the Eq. (7), we obtain a solution to forced Euler. Furthermore, if the $U_{k}$ are chosen appropriately, (6) can be solved explicitly. We can then use this simplified evolution equations to construct the blow-up.

Note that, *a priori*, the forces $F_{k}$ will have very bad regularity, and we can only hope that they belong to some well-posedness class if the approximations we have chosen for our evolution equation are very good. This is specially challenging considering the approximations need to remain valid along the whole evolution of the layers. Furthermore, since we are dealing with fractional Navier–Stokes, we also need to obtain useful approximations for $|\nabla |^{\alpha }\omega _{k}$, specifically approximations that still allow us to solve our simplified model explicitly, that do not worsen the other approximations we are considering, and that do not change the qualitative behaviour of our solutions.

#### Vortex Layers

Our blow-up solution consists of infinitely many vortex layers, each greater in magnitude, closer to the origin and existing for a shorter perioud than the previous one. Specifically, our vorticity $\omega$ is an infinite sum of $\omega _n$ which is nonzero only on the time interval $[T_n, 1]$. At the time of blow-up,^2^

8 $$\begin{aligned} \omega _n \approx A_n\psi (L_nx_1)\psi (L_nx_2)\psi (L_nx_3)\sin (M_nx_{n+2})\sin (M_nx_{n+3})e_{n+1}, \end{aligned}$$

where $A_n$, $M_n$ and $L_n \gg 1$, $\psi$ is a cut-off function and the subscripts are taken modulo 3. Then the velocity field generated by $\omega _0, \dots , \omega _{n-1}$, especially the one generated by $\omega _{n-1}$:

$$ \omega _{n-1} \approx A_{n-1}\psi (L_{n-1}x_1)\psi (L_{n-1}x_2)\psi (L_{n-1}x_3)\sin (M_{n-1}x_{n+1})\sin (M_{n-1}x_{n+2})e_n, $$

is roughly a constant times

$$\begin{aligned} U_n&\approx (A_{n-1}/M_{n-1})\psi (L_{n-1}x_1)\psi (L_{n-1}x_2)\psi (L_{n-1}x_3)\sin (M_{n-1}x_{n+1})\\&\quad \times \cos (M_{n-1}x_{n+2})e_{n+1}-(A_{n-1}/M_{n-1})\psi (L_{n-1}x_1)\psi (L_{n-1}x_2)\\&\quad \times \psi (L_{n-1}x_3)\cos (M_{n-1}x_{n+1})\sin (M_{n-1}x_{n+2})e_{n+2}. \end{aligned}$$

We will arrange that $L_n \gg L_{n-1}$, so $\omega _n$ is supported much closer to the origin than $\omega _{n-1}$. Then the velocity felt by $\omega _n$ has a first order approximation near the origin:

9 $$\begin{aligned} U_n \approx A_{n-1}x_{n+1}e_{n+1} - A_{n-1}x_{n+2}e_{n+2}. \end{aligned}$$

This is a saddle point, whose effect is stretching the $x_{n+1}$ direction while compressing the $x_{n+2}$ direction. Thanks to the vorticity stretching term $\omega \cdot \nabla u$ in the vorticity formulation (2), $\omega _n$, whose main term has been made to align with $e_{n+1}$, is thus stretched and leads to the blow-up.

#### Constraints on the Choice of the Parameters

The choice of the parameters are constrained by a number of factors. First, the stretching caused by the outer vortex layers to $\omega _n$ is of magnitude $A_{n-1}$. For the blow-up to happen, the stretching should beat the dissipation, which is of magnitude $M_n^\alpha$, so we have our first constraint:

$$ M_n^\alpha \le A_{n-1}. $$

We will switch on $\omega _n$ purely by force, and then amplify it by a factor of $K_n$ to a magnitude of $A_n$ at the time of blow-up using the velocity field generated by the outer vortex layers. Then when $\omega _n$ first appears, it has the magnitude of $A_n/K_n$. This amplification is related to the vortex-stretching effect of $\nabla U_n$, but $\nabla U_n$ also appears in the transport term, whose effect is compressing $\omega _n$ by a factor of $K_n$ in the $x_{n+2}$ direction, and expanding it by roughly the same factor in the $x_{n+1}$ direction. After this compression $\omega _n$ has frequency $M_n$ in both directions, so it initially has frequency $M_n/K_n$ in the $x_{n+2}$ direction, while its frequency in the $x_{n+3}$ direction is still almost $M_n$, so its $C^s$ norm is then $A_nM_n^s/K_n$, which should be at most of order 1, so our second constraint is

$$ A_nM_n^s/K_n \le 1. $$

In addition, for the approximation (9) to work, the support of $\omega _n$ should not exceed the wavelength of $\omega _{n-1}$, so that we can use the approximation $\sin x \sim x$ for the velocity field $U_n$ generated by the outer vortex layers. This should hold throughout the period when $\omega _n$ exists, especially right after it is turned on, when its support has size $K_n/L_n$ in the $x_{n+2}$ direction because this direction is compressed by a factor of $K_n$ to a final support of size $\frac{1}{L_n}$. Hence

$$ K_n/L_n \le 1/M_{n-1}. $$

Finally, when $$\omega _n$$ evolves, the main term of error comes from its self-interaction, which is corrected by force. Since $$\omega _n$$ has magnitude at most $$ A_n$$, the $$C^s$$ norm of the quadratic terms $$u \cdot \nabla \omega $$ and $$\omega \cdot \nabla u$$ are of sizes $$A_n^2M_n^s$$. However, the sinusodial shape of the vorticity makes main terms cancel out (see Lemma 7 for details). The error of the next order is smaller by a factor of $$M_n/L_n$$, because the frequency $$M_n$$ in the leading order is replaced by the frequency of the cutoff $$L_n$$. Then the error is of size $$A_n^2L_nM_n^{s-1}$$. Because the speed of amplification is $$A_{n-1}$$ per unit time, $$\omega _n$$ exists for a time period of $$(\ln K_n)/A_{n-1} \ge 1/A_{n-1}$$, so in order to bound the $$L_t^1C_x^s$$ norm of the error, we must have

$$ A_n^2L_nM_n^{s-1}/A_{n-1} \le 1. $$

Solving the constraints one by one, we get

$$ K_n \ge A_nM_n^s, \quad L_n \ge K_nM_{n-1} \ge A_nM_n^sM_{n-1}, $$

and finally,

$$ A_n^3M_n^{2s-1}M_{n-1} \le A_{n-1}. $$

It is now reasonable to take the equality in the constraint $M_n^\alpha \le A_{n-1}$. Then we get

$$ M_{n+1}^{3\alpha }M_n^{2s-1-\alpha }M_{n-1} \le 1. $$

or

10 $$\begin{aligned} 3\alpha \ln M_{n+1} + (2s-1-\alpha )\ln M_n + \ln M_{n-1} \le 0. \end{aligned}$$

This is a linear recurrence inequality in terms of $\ln M_n$. To saturate it, it makes sense to take $\ln M_n = R^n$, where *R* is a root of the characteristic equation.^3^ Since $M_n \gg 1$, $R > 0$. This requires the characteristic equation to have a positive root, so

$$ \Delta = (2s-1-\alpha )^2 - 12\alpha \ge 0,\quad 2s-1-\alpha <0, $$

giving

$$ s \le s(\alpha ):= (1 + \alpha )/2 - \sqrt{3\alpha }. $$

For such a choice of *s*, and with the dissipation $|\nabla |^\alpha$, the forcing upon $\omega _n$ can be controlled in the space $L_1^tC_x^{s(\alpha )-}$. Now all we need is $s(\alpha ) > 0$. Note that $s(0) = 1/2$, so we recover the $C^{1,\frac{1}{2}-}$ regularity for the Euler blow-up, obtained previously by two of the authors in [13]. When $\alpha$ is a small positive number, we still have $s(\alpha ) > 0$. Then the characteristic equation has two real roots whose product is $\frac{1}{3\alpha } > 1$ by Vieta’s theorem, so there is a root $R > 1$. We will morally take $M_n = R^n$ and saturate all the inequalities above, which then yield all the other parameters $A_n$, $K_n$ and $L_n$ one by one. At the time of blow-up, the Hölder regularity of the velocity is $C^{1-\log _{M_n}{A_n}}$, which will turn out to be $C^{1-\sqrt{2\alpha /7}}$ (see the choice of parameters at the beginning of Section 4). As a concrete example, when $\alpha = 1/14$ one can take $R = 2$, $M_n = N^{2^n}$, $A_n = M_n^{1/7}$, $M_n = M_n^{1/7+s}$ and $L_n = M_n^{11/14-s}$, which work for *s* small. Note that this is only a back-of-the-envelope calculation: in reality there are other sources of error getting in the way.

#### Symmetry of the Construction

In our construction, the vorticity and the velocity it generates satisfies certain symmetry with respect to reflections across coordinate planes. We say that a vector field *V*(*x*) is **polar** if for all $j = 1, 2, 3$, $V_j(x)$ is odd in $x_j$, and that *V*(*x*) is **axial** if for all $j = 1, 2, 3$, $V_j(x)$ is even in $x_j$ but odd in $x_k$ for all $k \ne j$. The vorticity of our blow-up will thus be axial and by the Biot–Savart law, the velocity it generates is polar.

### Outline of the Paper

In Section 2 we study how the flow generated by (a more delicate version of) (9) deforms the vortex layer $\omega _n$. In Section 3, we estimate how this deformation introduces various errors in the linear and bilinear terms. In Section 4, we spell out the construction itself and show that all the errors are under control.

## Flow Map Estimates

The velocity *U* that the outer vortex layers generate is polar, meaning that $U_j$ is odd in $x_j$ ($j = 1, 2, 3$), but we will not use *U* itself, but the following approximation to generate the flow that transports the inner vortex layers:

$$ (x_1\partial _1U_1(0, x_2, 0, t), U_2(0, x_2, 0, t), x_3\partial _3U_3(0, x_2, 0, t)). $$

In this section we study the flow generated by this field. Note that the $e_2$ component only depends on $x_2$, and the $e_j$ component ($j = 1, 3$) is linear in $x_j$; thus the flow can be solved explicitly, first in the $e_2$ component, then in the other components. This special structure of the velocity will give us useful properties of the flow map, that will be crucial for our construction. In particular, it will ensure that the frequency of our sinusoids changes slowly in space.

### Lemma 1

Let *u*(*x*, *t*) be a smooth scalar function that is odd in *x*. Assume that $X \ge 0$ and that for all $t \ge 0$, $X^2\sup |\partial _x^3u(\cdot , t)| \le -\partial _xu(0, t)$. Then for $|x| \le X$, the solution to

$$ \phi (x, 0) = x, \quad \partial _t\phi (x, t) = u(\phi (x, t), t) $$

exists for all $t \ge 0$ and satisfies that

(i) $$|\phi (x, t)| \le |x|$$.

(ii) $\phi (x, t)$ has the sign of *x*.

(iii) $$|\phi (x, t)| \le |x|\exp \int _0^t (\partial _xu(0, s) + \frac{x^2\sup |\partial _x^3u(\cdot , s)|}{6})\text {d}s$$.

(iv) $$|\phi (x, t)| \ge |x|\exp \int _0^t (\partial _xu(0, s) - \frac{x^2\sup |\partial _x^3u(\cdot , s)|}{6})\text {d}s$$.

(v) $$\partial _x\phi (x, t) \le \exp \int _0^t (\partial _xu(0, s) + \frac{x^2\sup |\partial _x^3u(\cdot , s)|}{2})\text {d}s \le 1$$.

(vi) $$\partial _x\phi (x, t) \ge \exp \int _0^t (\partial _xu(0, s) - \frac{x^2\sup |\partial _x^3u(\cdot , s)|}{2})\text {d}s$$.

(vii) $$|\partial _{xx}\phi (x, t)| \le |x|\int _0^t \sup |\partial _x^3u(\cdot , s)|\text {d}s$$.

### Proof

(i) For $x = 0$, the solution exists and equals 0 for all time. Now we suppose that $x \ne 0$. Let *I* be the maximal interval in $\mathbb {R}^+$ on which the solution exists and stays in $(-2|x|,2|x|) \subset (-2X, 2X)$. Since $\phi (x, 0) = x$, by continuity, *I* is non-empty. For $t \in I$, by Taylor’s theorem with the Lagrange remainder and the hypothesis on $\sup |\partial _1^3u(\cdot , t)|$,

$$\begin{aligned} |u(\phi (x, t), t) - \partial _xu(0, t)\phi (x, t)|&\le \frac{|\phi (x, t)|^3}{6}\sup |\partial _x^3u(\cdot , t)|\\&\le \frac{|\phi (x, t)|^3}{6}\frac{|\partial _xu(0, t)|}{X^2} \le \frac{2|\partial _xu(0, t)\phi (x, t)|}{3}, \end{aligned}$$

so $\partial _t\phi (x, t) = u(\phi (x, t), t)$ has the sign of $\partial _xu(0, t)\phi (x, t)$, or that of $-\phi (x, t)$ (because $\partial _xu(0, t) \le 0$), so $|\phi (x, t)| \le |\phi (0, t)| \le |x|$. If $I \ne \mathbb {R}$, then by local existence, the solution can be extended beyond *I* while still staying in $(-2|x|,2|x|)$. Hence the solution exists for all $t \ge 0$, and satisfies $|\phi (t)| \le |x|$.

(ii) Since *u* is smooth, by the uniqueness theorem, any solution with $x \ne 0$ does not reach 0, so by continuity, $\phi (t)$ has the sign of *x*.

(iii) and (iv) By Taylor’s theorem with the Lagrange remainder,

$$\begin{aligned} |u(\phi (x, t), t) - \partial _xu(0, t)\phi (x, t)|&\le \frac{|\phi (x, t)|^3\sup |\partial _x^3u(\cdot , t)|}{6}\\&\le \frac{x^2\sup |\partial _x^3u(\cdot , t)|}{6}|\phi (x, t)|. \end{aligned}$$

Multiplication by $2|\phi (x, t)|$ gives

$$ |\partial _t(\phi (x, t)^2) - 2\partial _xu(0, t)\phi (x, t)^2| \le \frac{x^2\sup |\partial _x^3u(\cdot , t)|}{3}\phi (x, t)^2 $$

so

$$ \partial _t(\phi (x, t)^2) \le (2\partial _xu(0, t) + \frac{x^2\sup |\partial _x^3u(\cdot , t)|}{3})\phi (x, t)^2 $$

so

$$ \phi (x, t)^2 \le x^2\exp \int _0^t (2\partial _xu(0, s) + \frac{x^2\sup |\partial _x^3u(\cdot , s)|}{3})\text {d}s $$

so

$$ |\phi (x, t)| \le |x|\exp \int _0^t (\partial _xu(0, s) + \frac{x^2\sup |\partial _x^3u(\cdot , s)|}{6})\text {d}s. $$

The inequality in the other direction follows similarly.

(v) and (vi) Taking the *x* derivative of the ODE gives

$$ \partial _{tx}\phi (x, t) = \partial _xu(\phi (x, t), t)\partial _x\phi (x, t). $$

Since $$\partial _x\phi (x, 0) = 1$$,

$$ \partial _x\phi (x, t) = \exp \int _0^t \partial _xu(\phi (x, s), s)\text {d}s. $$

By Taylor’s theorem with the Lagrange remainder,

$$ |\partial _xu(\phi (x, t), t) - \partial _xu(0, t)| \le \frac{|\phi (x, t)|^2\sup |\partial _x^3u(\cdot , t)|}{2} \le \frac{x^2\sup |\partial _x^3u(\cdot , t)|}{2}, $$

so

$$ \left| \int _0^t \partial _xu(\phi (x, s), s)\text {d}s - \int _0^t \partial _xu(0, s)\text {d}s \right| \le \frac{x^2}{2}\int _0^t \sup |\partial _x^3u(\cdot , s)|\text {d}s, $$

which gives the first inequality of (v), as well as (vi). For the second inequality of (v), Note that $x^2|\partial _x^3u(\cdot , t)| \le |\partial _xu(0, t)|$, so $\partial _xu(0, s) + \frac{x^2\sup |\partial _x^3u(\cdot , s)|}{2}$ has sign of $\partial _1u(0, s) \le 0$.

(vii) Taking another *x* derivative of the ODE gives

$$ \partial _{txx}\phi (x, t) = \partial _x^2u(\phi (x, t), t)\partial _x\phi (x, t)^2 + \partial _xu(\phi (x, t), t)\partial _{xx}\phi (x, t). $$

Since

$$ |\partial _xu(\phi (x, t), t) - \partial _xu(0, t)| \le \frac{x^2\sup |\partial _x^3u(\cdot , t)|}{2} \le \frac{\partial _xu(0, t)}{2}, $$

$\partial _xu(\phi (x, t), t)$ has the sign of $\partial _xu(0, t)$, which in non-positive. Then $\partial _xu(\phi (x, t), t)\partial _{xx}\phi (x, t)$ has the sign of $-\partial _{xx}\phi (x, t)$, so

$$ \partial _t|\partial _{xx}\phi (x, t)| \le \partial _x^2u(\phi (x, t), t)\partial _x\phi (x, t)^2. $$

Using $|\partial _x^2u(\phi (x, t), t)| \le |x|\sup |\partial _x^3u(\cdot , t)|$ and (v) gives

$$ \partial _t|\partial _{xx}\phi (x, t)| \le |x|\sup |\partial _x^3u(\cdot , t)|. $$

Since $\partial _{xx}\phi (x, 0) = 0$, (vii) follows. $\square$

### Lemma 2

Let *u*(*x*, *t*) be a smooth scalar function that is odd in *x*. Let $\phi (x, t_0, t)$ be its flow map, satisfying

$$ \phi (x, t_0, t_0) = x, \quad \partial _t\phi (x, t_0, t) = u(\phi (x, t_0, t)). $$

Let

$$ K(t) = \exp \int _t^1 -\partial _xu(0, s)\text {d}s. $$

Let $t \le 1$ and $X \ge 0$. Assume for all $s \in [t, 1]$, $X^2\sup |\partial _x^3u(\cdot , s)| \le -\partial _xu(0, s)$. Then

(i) For $$|x| \le K(t)^{-1}X\exp \int _t^1 -\frac{X^2\sup |\partial _x^3u(\cdot , s)|}{6}\text {d}s$$, $$|\phi (x, 1, t)| \le X$$.

(ii) For $$|x| \le X$$, $$\partial _x\phi (x, t, 1) \le K(t)^{-1}\exp \int _t^1 \frac{x^2\sup |\partial _x^3u(\cdot , s)|}{2}\text {d}s \le 1$$.

(iii) For $$|x| \le X$$, $$|\partial _x^2\phi (x, t, 1)| \le |x|\int _t^1 \sup |\partial _x^3u(\cdot , s)|\text {d}s$$.

### Proof

(i) Assume that $|\phi (x, 1, t)| > X$, or that the flow map cannot be extended to time *t*. Since $\partial _xu(0, s) \le 0$, $K(t) \ge 1$, so $|\phi (x, 1, 1)| = |x| \le X$. Then there is $t < t_1 \le 1$ such that $|\phi (x, 1, t_1)| = X$. Since

$$\begin{aligned} \phi (\phi (x, 1, t_1), t_1, t_1)&= \phi (x, 1, t_1) = \pm X,\\ \partial _t\phi (\phi (x, 1, t_1), t_1, t)&= u(\phi (\phi (x, 1, t_1), t_1, t), t), \end{aligned}$$

$\phi (\phi (x, 1, t_1), t_1, t)$ satisfies the ODE in Lemma 1, with initial data $\pm X$ satisfying the assumption. Then, by Lemma 1 (iv),

$$\begin{aligned} |\phi (\phi (x, 1, t_1), t_1, 1)|&\ge K(t_1)^{-1}X\exp \int _{t_1}^1 -\frac{X^2\sup |\partial _x^3u(\cdot , s)|}{6}\text {d}s\\&\ge K(t)^{-1}X\exp \int _t^1 -\frac{X^2\sup |\partial _x^3u(\cdot , s)|}{6}\text {d}s. \end{aligned}$$

By the reversibility of the flow, $\phi (\phi (x, 1, t_1), t_1, 1) = x$. Hence the inequality on *x* in (i) is saturated. If $X = 0$ then $x = 0$, so $\phi (x, 1, s) = 0$ by the uniqueness theorem. Otherwise $K(t) = K(t_1)$, so for all $s \in [t, t_1]$, $\partial _xu(0, s) = 0$. By the assumption, $\sup |\partial _x^3u(\cdot , s)| = 0$. Since *u* is odd in *x*, $u(\cdot , s) \equiv 0$ for all $s \in [t, t_1]$, so $|\phi (x, 1, t)| = |\phi (x, 1, t_1)| = X$, a contradiction.

(ii) and (iii) follow from Lemma 1 (v) and (vii) respectively. $\square$

Now we solve the flow in the remaining components.

### Lemma 3

Let *U*(*x*, *t*) be a smooth, polar and divergence-free velocity field on $\mathbb {R}^3$ of the form

$$ U(x, t) = (x_1\partial _1U_1(0, x_2, 0, t), U_2(0, x_2, 0, t), x_3\partial _3U_3(0, x_2, 0, t)). $$

Let $\phi (x, t_0, t)$ be its flow map, satisfying

$$ \phi (x, t_0, t_0) = x, \quad \partial _t\phi (x, t_0, t) = U(\phi (x, t_0, t)). $$

Let

$$ K = \exp \int _t^1 -\partial _2U_2(0, s)\text {d}s. $$

Let $t \le 1$. Assume for all $s \in [t, 1]$, $X^2\sup |\partial _2^3U_2(0, \cdot , 0, s)| \le -\partial _2U_2(0, 0, 0, s)$. Then for $|x_2| \le X$,

$$ \phi _1(x, t, 1) = x_1K\psi _1(x_2), \quad \phi _3(x, t, 1) = x_3\psi _3(x_2), $$

where

$$\begin{aligned} |\ln \psi _3(x_2)|&\le \int _t^1 \sup |\partial _3U_3(0, \cdot , 0, s)|\text {d}s,\\ |\ln \psi _1(x_2)|&\le \int _t^1 (\sup |\partial _3U_3(0, \cdot , 0, s)| + \frac{x_2^2\sup |\partial _2^3U_2(0, \cdot , 0, s)|}{2})\text {d}s,\\ |(\ln \psi _3)'(x_2)|&\le |x_2|\int _t^1 \sup |\partial _{223}U_3(0, \cdot , 0, s)|\text {d}s,\\ |(\ln \psi _1)'(x_2)|&\le |x_2|\int _t^1 (\sup |\partial _{223}U_3(0, \cdot , 0, s)| + \sup |\partial _2^3U_2(0, \cdot , 0, s)|)\text {d}s,\\ |(\ln \psi _3)''(x_2)|&\le (1 + \ln K)\int _t^1 \sup |\partial _{223}U_3(0, \cdot , 0, s)|\text {d}s,\\ |(\ln \psi _1)''(x_2)|&\le (1 + \ln K)\int _t^1 (\sup |\partial _{223}U_3(0, \cdot , 0, s)| + \sup |\partial _2^3U_2(0, \cdot , 0, s)|)\text {d}s. \end{aligned}$$

### Remark 2

$\psi _1$ and $\psi _3$ also depend on *t*, but like $\phi$ we will only focus on its derivative in $x_2$, so we suppress the dependence on *t* to simplify the notation.

### Proof

We have

$$\begin{aligned} |\ln \psi _3(x_2)|&= |\int _t^1 \partial _3U_3(0, \phi _2(x, t, s), 0, s)\text {d}s| \le \int _t^1 \sup |\partial _3U_3(0, \cdot , 0, s)|\text {d}s,\\ \ln \psi _1(x_2)&= \int _t^1 (\partial _1U_1(0, \phi _2(x, t, s), 0, s) + \partial _2U_2(0, s))\text {d}s\\&= \int _t^1 (\partial _2U_2(0, s) - \partial _2U_2(0, \phi _2(x, t, s), 0, s))\text {d}s - \ln g_3(x_2). \end{aligned}$$

By Lemma 1 (i), $|\phi _2(x, t, s)| \le |x_2|$, so by Taylor’s theorem with the Langrange remainder,

$$ |\partial _2U_2(0, s) - \partial _2U_2(0, \phi _2(x, t, s), 0, s)| \le \frac{x_2^2\sup |\partial _2^3U_2(0, \cdot , 0, s)|}{2} $$

so

$$ |\ln \psi _1(x_2)| \le \int _t^1 (\sup |\partial _3U_3(0, \cdot , 0, s)| + \frac{x_2^2\sup |\partial _2^3U_2(0, \cdot , 0, s)|}{2})\text {d}s. $$

Also,

$$\begin{aligned} |(\ln \psi _3)'(x_2)|&= |\int _t^1 \partial _{23}U_3(0, \phi _2(x, t, s), 0, s)\partial _2\phi _2(x, t, s)\text {d}s|\\&\le \int _t^1 |\partial _{23}U_3(0, \phi _2(x, t, s), 0, s)|\text {d}s \quad \qquad {(\textrm{by} \quad \textrm{Lemma} \quad 2 \quad \mathrm{(ii)})}\\&\le |x_2|\int _t^1 \sup |\partial _{223}U_3(0, \cdot , 0, s)|\text {d}s, \quad \qquad {(\partial _{23}U_3(0, s) = 0)}\\ |(\ln \psi _1)'(x_2)|&\le \int _t^1 |\partial _2^2U_2(0, \phi _2(x, t, s), 0, s)\partial _2\phi _2(x, t, s|)\text {d}s + |(\ln g_3)'(x_2)|\\&\le |x_2|\int _t^1 (\sup |\partial _{223}U_3(0, \cdot , 0, s)| + \sup |\partial _2^3U_2(0, \cdot , 0, s)|)\text {d}s. \end{aligned}$$

Also,

$$\begin{aligned}&|(\ln \psi _3)''(x_2)| \\  &\quad \le \int _t^1 |\partial _{223}U_3(0, \phi _2(x, t, s), 0, s)|\text{ d }s \quad {(\text {by}\quad \text {Lemma } \,\,2 \quad \mathrm {(ii)})}\\  &\quad \quad + \int _t^1 |\partial _{23}U_3(0, \phi _2(x, t, s), 0, s)\partial _2^2\phi _2(x, t, s)|\text{ d }s\\  &\quad \le \int _t^1 \sup |\partial _{223}U_3(0, \cdot , 0, s)|\text{ d }s\\  &\quad \quad + X^2\int _t^1 \sup |\partial _{223}U_3(0, \cdot , 0, s)|\text{ d }s\int _t^1 \sup |\partial _2^3U_2(0, \cdot , 0, s)|\text{ d }s \quad {(\text {by } \quad \text {Lemma } \quad 2 \quad \mathrm {(iii)})}\\  &\quad \le \int _t^1 \sup |\partial _{223}U_3(0, \cdot , 0, s)|\text{ d }s\left( 1 - \int _t^1 \partial _2U_2(0, s)\text{ d }s\right) \quad \quad {(\text {by} \quad \text {assumption})}\\  &\quad = (1 + \ln K)\int _t^1 \sup |\partial _{223}U_3(0, \cdot , 0, s)|\text{ d }s. \end{aligned}$$

Similarly,

$$\begin{aligned} |(\ln \psi _1)''(x_2)|&\le \int _t^1 |\partial _2^3U_2(0, \phi _2(x, t, s), 0, s)|\text {d}s\\&+ \int _t^1 |\partial _2^2U_2(0, \phi _2(x, t, s), 0, s)\partial _2^2\phi _2(x, t, s)|\text {d}s + |(\ln g_3)''(x_2)|\\&\le (1 + \ln K)\int _t^1 (\sup |\partial _{223}U_3(0, \cdot , 0, s)| + \sup |\partial _2^3U_2(0, \cdot , 0, s)|)\text {d}s. \end{aligned}$$

$$\square $$

The behavior of the vortex layer transported by *U* can now be studied. We also include a term that corresponds to the dissipation.

### Lemma 4

Let *U*, $\phi$, *K*, $\psi _1$ and $\psi _3$ be as in Lemma 3. For $i = 1, 3$, let $\omega$ be a smooth and axial vector field on $\mathbb {R}^3$. Let *g* be a function of *t* only. Then the solution to the PDE

$$ \partial _t\omega _i + U \cdot \nabla \omega _i = \omega _i\partial _iU_i + g\omega _i $$

is

$$\begin{aligned} \omega _1(x, t)&= (GK)^{-1}\psi _1(x_2)^{-1}\omega _1(\phi (x, t, 1), 1),\\ \omega _3(x, t)&= G^{-1}\psi _3(x_2)^{-1}\omega _3(\phi (x, t, 1), 1) \end{aligned}$$

where

$$ G = e^{\int _t^1\,g(s)\text {d}s}. $$

### Proof

Using $\partial _t(G\omega _i) = G(\partial _t\omega _i - g\omega _i)$ we can assume that $g = 0$ and $G = 1$.

Since $\omega _i$ is odd in $x_i$, it vanishes when $x_i = 0$. Since it is also smooth, we can put $\omega _i = x_i\omega _i^\circ$, where $\omega _i^\circ$ is smooth. Then

$$ \partial _t\omega _i + U \cdot \nabla \omega _i = x_i\partial _t\omega _i^\circ + x_iU \cdot \nabla \omega _i^\circ + \omega _i^\circ U \cdot \nabla x_i = x_i\partial _t\omega _i^\circ + x_iU \cdot \nabla \omega _i^\circ + \omega _i\partial _iU_i $$

where we have used $\omega _i^\circ U \cdot \nabla x_i = \omega _i^\circ U_i = \omega _i^\circ x_i\partial _iU_i = \omega _i\partial _iU_i$. After cancelling this term from both sides of the PDE we see that $\omega _i^\circ = \omega _i/x_i$ is conserved along the flow line. Thus

$$\begin{aligned} \omega _1(x, t)&= x_1\omega _1(\phi (x, t, 1), 1)/\phi _1(x, t, 1) = K^{-1}\psi _1(x_2)^{-1}\omega _1(\phi (x, t, 1), 1),\\ \omega _3(x, t)&= x_3\omega _3(\phi (x, t, 1), 1)/\phi _3(x, t, 1) = \psi _3(x_2)^{-1}\omega _3(\phi (x, t, 1), 1). \end{aligned}$$

$$\square $$

## Velocity Estimates

Let $\psi$ be a smooth and even bump function supported in $[-1, 1]$. We first prescribe one component of the vorticity (with amplitude normalized to $\sim 1$),

$$\begin{aligned} \omega _1&= a(x_2)\psi (L_1g_1(x_2)x_1)\sin (Mg_2(x_2))\psi (L_2g_2(x_2))\\&\quad \sin (Mg_3(x_2)x_3)\psi (L_3g_3(x_2)x_3), \end{aligned}$$

(actually $a = 1/g_1$, but this generality is useful later) and estimate the velocity (that of its derivatives) it generates, which we write as $\omega * \mathcal {K}$, where $\mathcal {K}$ is the Biot–Savart kernel.

Throughout this section we assume that $M \ge 1$, $L_1, L_2, L_3 \in [1, M^{1-\delta }]$ ($0 < \delta \le 1$), $|a| \le 2$, $g_1(x_2), g_3(x_2) \in [1/2, 2]$ and $|g_2'(x_2)| \le 1$, unless stated otherwise.

For any multi-index $J = (j_1, j_2, j_3)$ let $|J| = \sum _{i=1}^3 j_i$ and $\partial _J = \partial _1^{j_i}\partial _2^{j_2}\partial _3^{j_3}$.

Let $\delta (j) = 1$ if $j = 0$ and 0 if $j > 0$. We will approximate the velocity $\omega * \mathcal {K}$ (and its derivatives of order *J*) generated by $\omega _1$ using $\tilde{u} = \tilde{u}_{,0}$ and $\tilde{u}_{,J} = (0, \tilde{u}_{2,J}, \tilde{u}_{3,J})$, where

$$\begin{aligned} \tilde{u}_{2,J}&= \frac{\delta (j_1)g_2'(x_2)^{j_2}g_3(x_2)^{1+j_3}a(x_2)\psi (L_1g_1(x_2)x_1)\sin (Mg_2(x_2) + j_2\pi /2)}{M^{1-|J|}(g_3(x_2)^2 + g_2'(x_2)^2)}\\&\quad \times \psi (L_2g_2(x_2))\cos (Mg_3(x_2)x_3 + j_3\pi /2)\psi (L_3g_3(x_2)x_3),\\ \tilde{u}_{3,J}&= -\frac{\delta (j_1)g_2'(x_2)^{1+j_2}g_3(x_3)^{j_3}a(x_2)\psi (L_1g_1(x_2)x_1)\cos (Mg_2(x_2) + j_2\pi /2)}{M^{1-|J|}(g_3(x_2)^2 + g_2'(x_2)^2)}\\&\quad \times \psi (L_2g_2(x_2))\sin (Mg_3(x_2)x_3 + j_3\pi /2)\psi (L_3g_3(x_2)x_3). \end{aligned}$$

This approximation is obtained by treating $\omega$ as a planar sine wave (effectively taking $L_i = 0$), on which the action of the Biot–Savart kernel, as a Fourier multiplier, is exactly scalar multiplication. At the other extreme, if $L_i = M$, then there is only one wave length in the support in each direction, so the wave form could deviate significantly from that of a planar sine wave, so the error would be as large as the main term, i.e., of order 1. For intermediate values of $L_i$, we would thus expect an error of order $\sum L_i/M \ll 1$, plus some more error coming from the deformation caused by the flow (evidenced in the non-constancy of $g_i$), which will turn out to be harmless.

### Linear Estimates

Since $\omega$ is of order 1, $\omega * \mathcal {K}$ is of order 1/*M*, so $\partial _J(\omega * \mathcal {K})$ is of order $M^{|J|-1}$. Hence we expect an error of order $\sum L_iM^{|J|-2}$ in the approximation, which is the content of the following lemma.

#### Lemma 5

For $$|J| \le 1$$,

$$ |\tilde{u}_{,J} - \partial _J(\omega _1e_1 * \mathcal {K})| \lesssim _\delta BM^{(|J|-2)(1-\delta )}, $$

where $$B = \sum _{i=1}^3 L_i + L_3\ln M + \sup |a'| + \sup |g_1'| + ML_3^{-1}(\sup |g_3'| + \sup |g_2''|)$$.

#### Proof

We can decompose all factors of the form $\sin X$ as a linear combination of $e^{\pm iX}$ and $\cos X$ as a linear combination of $e^{\pm i(X + \pi /2)} = \pm ie^{\pm iX}$ respectively, so we can replace all factors of the form $\sin X$ with $e^{iX}$ and $\cos X$ with $e^{i(X + \pi /2)} = ie^{iX}$ respectively, in both $\omega _1$ and $\tilde{u}_{,J}$. We then have

$$\begin{aligned} \partial _J(\omega _1e_1 * \mathcal {K})&= \int _{\mathbb {R}^3} \frac{h\times e_1}{4\pi |h|^3}\partial _J\omega _1(x + h)\text {d}h\\&= (-1)^{|J|}PV\int _{\mathbb {R}^3} \omega _1(x + h)\partial _J\frac{h\times e_1}{4\pi |h|^3}\text {d}h + c_J\omega _1(x), \end{aligned}$$

where $c_{(0,0,0)} = 0$, and if $J_j = 1$, $c_J$ is a constant vector depending only on *J* because the last term comes from the boundary term $-\lim _{r\rightarrow 0}\int _{S(r)} \frac{h\times e_1}{4\pi |h|^3}\omega _1(x+h)d\textbf{S}\cdot e_j =-\omega _1(x)\int _{S(1)} \frac{h\times e_1}{4\pi }h_j\text {d}S$.

Integrating by parts in $h_3$ we get, for any $k \in \mathbb {N}$, $n \!=\! 3, 5$ and $m \!=\! 0, 1, \dots , n \!-\! 2$,

$$\begin{aligned}&\left| \int _{\mathbb {R}} \frac{h_3^me^{iMg_3(x_2 + h_2)(x_3 + h_3)}\psi (L_3g_3(x_2 + h_2)(x_3 + h_3))}{|h|^n}\text {d}h_3 \right| \\&= \left| \int _{\mathbb {R}} \frac{i^ke^{iMg_3(x_2 + h_2)(x_3 + h_3)}}{M^kg_3(x_2 + h_2)^k} \frac{\partial ^k}{\partial h_3^k}\frac{h^m\psi (L_3g_3(x_2 + h_2)(x_3 + h_3))}{|h|^n}\text {d}h_3 \right| \\&\lesssim _k M^{-k}\sum _{j=0}^k L_3^j\int _{\mathbb {R}} |h|^{m-n-k+j}\text {d}h_3 \lesssim _k M^{-k}\sum _{j=0}^k L_3^j(h_1^2 + h_2^2)^{-(n-m-1+k-j)/2} \end{aligned}$$

Since each component of $\partial _J(h/|h|^3)$ is of the form $P(h)/|h|^{2|J|+3}$ where *P* is a homogeneous polynomial of degree $|J| + 1$, we can apply the above with $n = 2|J| + 3$ and $m = \deg _3P \le |J| + 1 \le n - 2$. After including $|J| + 1 - m$ factors of $h_1$ and $h_2$ we get

$$\begin{aligned}&\left| \int _{h_1^2+h_2^2\ge M^{-2+2\delta }} \omega _1(x + h)\partial _J\frac{h\times e_1}{|h|^3}\text {d}h \right| \\&\lesssim _k M^{-k}\sum _{j=0}^k L_3^j\int _{h_1^2+h_2^2\ge M^{-2+2\delta }\atop |x+h|\le 1} (h_1^2 + h_2^2)^{-(|J|+1+k-j)/2}\text {d}h_1\text {d}h_{2}, \end{aligned}$$

because $\omega$ is supported in $\{|x| \le 1\}$. Since this domain has area at most $\pi$, the right-hand side

$$\begin{aligned}&\lesssim _k M^{|J|-k}\sum _{j=0}^{k-2} L_3^jM^{(1-\delta )(k-j-1)} + M^{|J|-k}(L_3^{k-1}\ln M + L_3^k)\\  &\lesssim _k M^{|J|-\delta k}(1 + M^{-1+\delta }\ln M) \lesssim _\delta M^{-2} {(k := \left\lfloor \frac{|J| + 2}{\delta }\right\rfloor + 1)}, \end{aligned}$$

so if we divide $\omega =\chi (M^{1-\delta }h)\omega +(1-\chi (M^{1-\delta }h))\omega$, where $\chi (h) = \chi (h_1, h_2)$ is a smooth bump function equal to 1 near the origin, we can use the previous bounds to obtain good estimates for the contribution coming from $(1-\chi (M^{1-\delta }h))\omega$.

Denote $f(a) - f(b)$ by $f|_a^b$. Since $g_1 \le 2$ and $|J| \le 1$,

$$\begin{aligned}&\left| \int \chi (M^{1-\delta }h)\omega _1|_{(x_1, x_2 + h_2, x_3 + h_3)}^{x + h}\partial _J\frac{h \times e_1}{4\pi |h|^3}\text {d}h \right| \\&\quad \lesssim \int \chi (M^{1-\delta }h)\frac{\left| \omega _1|_{(x_1, x_2 + h_2, x_3 + h_3)}^{x + h}\right| }{|h|^{2+|J|}}\text {d}h\\&\quad \lesssim L_1\int \frac{\chi (M^{1-\delta }h)|h_1|}{|h|^{2+|J|}}\text {d}h_3\text {d}h_1\text {d}h_2\\&\quad \lesssim L_1\int \frac{\chi (M^{1-\delta }h)|h_1|\text {d}h_1\text {d}h_2}{(h_1^2 + h_2^2)^{(1+|J|)/2}},\\&\quad \lesssim L_1M^{(|J|-2)(1-\delta )} \end{aligned}$$

so we can replace $\omega _1(x + h)$ by $\omega _1(x_1, x_2 + h_2, x_3 + h_3)$.

Note that when computing $\partial _2(a(x_2)\psi (L_1g_1x_1)\psi (L_2g_2)\psi (L_3g_3(x_3 + h_3)) e^{iMg_3(x_2)(x_3 + h_3)})$, the following factors appear:

**Table:** 

Factor	Bound	$x_2$ derivative bound
$a(x_2)$	2	$\sup |a'|$
$\psi (L_1g_1(x_2)x_1)$	$\lesssim 1$	$\lesssim \sup |g_1'|$ ($|L_1g_1x_1| \lesssim 1$, so $L_1|x_1| \lesssim 1$)
$\psi (L_2g_2(x_2)$	$\lesssim 1$	$\lesssim L_2$ (note that $|g_2'| \le 1$)
$\psi (L_3g_3(x_2)(x_3 + h_3))$	$\lesssim 1$	$\lesssim \sup |g_3'|$ (similarly $L_3|x_3 + h_3| \lesssim 1)$
$e^{iMg_3(x_2)(x_3 + h_3)}$	1	$\lesssim ML_3^{-1}\sup |g_3'|$ ($M|x_3 + h_3| \lesssim ML_3^{-1})$
The product	$\lesssim 1$	$\lesssim B$ (we have used $M/L_3 \ge 1$)

Also, on the support of $\chi (M^{1-\delta }h),$ we have

$$\begin{aligned} \left| \exp |_{iM(g_2(x_2) + h_2g_2'(x_2))}^{iMg_2(x_2 + h_2)}\right|&\le Mh_2^2\sup |g_2''| < M^\delta |h_2|\sup |g_2''|\\  &\le ML_3^{-1}|h_2|\sup |g_2''| \le B|h_2|, \end{aligned}$$

so

$$\begin{aligned}&|\omega _1(x_1, x_2 + h_2, x_3 + h_3) + a(x_2)\psi (L_1g_1(x_2)x_1)e^{iM(g_2(x_2) + h_2g_2'(x_2))}\psi (L_2g_2(x_2))\\&\times e^{iMg_3(x_2)(x_3 + h_3)}\psi (L_3g_3(x_2)(x_3 + h_3))| \lesssim B|h_2| \end{aligned}$$

and

$$\begin{aligned}&\int \frac{\chi (M^{1-\delta }h)}{|h|^{2+|J|}}|\omega _1(x_1, x_2 + h_2, x_3 + h_3) - a(x_2)\psi (L_1g_1(x_2)x_1)e^{iM(g_2(x_2) + h_2g_2'(x_2))}\\&\times \psi (L_2g_2(x_2))e^{iMg_3(x_2)(x_3 + h_3)}\psi (L_3g_3(x_2)(x_3 + h_3))|\text {d}h\\&\lesssim B\int \frac{\chi (M^{1-\delta }h)|h_2|}{|h|^{2+|J|}}\text {d}h_3\text {d}h_1\text {d}h_2 \lesssim B\int \frac{\chi (M^{1-\delta }h)|h_2|\text {d}h_1\text {d}h_2}{(h_1^2+h_2^2)^{(1+|J|)/2}}\\&\lesssim BM^{(|J|-2)(1-\delta )}. \end{aligned}$$

Since $$g_3 \le 2$$, $$|\psi |_{L_3g_3(x_2)x_3}^{L_3g_3(x_2)(x_3 + h_3)}| \lesssim L_3|h_3|$$. Then

$$\begin{aligned}&\int \frac{\chi (M^{1-\delta }h)}{|h|^{2+|J|}}|a(x_2)\psi (L_1g_1(x_2)x_1)e^{iM(g_2(x_2) + h_2g_2'(x_2))}\psi (L_2g_2(x_2))e^{iMg_3(x_2)(x_3 + h_3)}\\&\times \psi |_{L_3g_3(x_2)x_3}^{L_3g_3(x_2)(x_3 + h_3)}|\text {d}h \lesssim L_3\int \int _{|x_3+h_3|\le 1} \frac{\chi (M^{1-\delta }h)|h_3|}{|h|^{2+|J|}}\text {d}h_3\text {d}h_1\text {d}h_2. \end{aligned}$$

because $\omega _1$ is supported in $\{|x| \le 1\}$. Note that the integral in $h_3$ is restricted to an interval of length *O*(1). If $|h_3| \ge 1$ then the integrand is also *O*(1), so the right-hand side

$$\begin{aligned}&\lesssim L_3M^{-2+2\delta } + L_3\int \int _0^1 \frac{\chi (M^{1-\delta }h)h_3\text {d}h_3}{|h|^{2+|J|}}\text {d}h_1\text {d}h_2\\&\lesssim L_3M^{-2+2\delta } + {\left\{ \begin{array}{ll} L_3\int \chi (M^{1-\delta }h)\ln \frac{h_1^2 + h_2^2 + 1}{h_1^2 + h_2^2}\text {d}h_1\text {d}h_2, &  |J|=0,\\ L_3\int \chi (M^{1-\delta }h)(h_1^2 + h_2^2)^{-1/2}\text {d}h_1\text {d}h_2, &  |J|=1 \end{array}\right. } \\&\lesssim L_3M^{(|J|-2)(1-\delta )}(1 + \ln M) \le BM^{(|J|-2)(1-\delta )}. \end{aligned}$$

Since the factors $a(x_2)\psi (L_1g_1(x_2)x_1)$, $e^{iMg_2(x_2)}$, $\psi (L_2g_2(x_2))$, $e^{iMg_3(x_2)x_3}$ and $\psi (L_3g_3(x_2)x_3)$ are independent of *h* and *O*(1), it suffices to show that

$$\begin{aligned}&\int \chi (M^{1-\delta }h)e^{iMg_2'(x_2)h_2}e^{iMg_3(x_2)h_3}(-1)^{|J|}\partial _J\frac{h\times e_1}{4\pi |h|^3}\text {d}h + c_J \\&\quad = \frac{i^{|J|+1}\delta (j_1)g_2'(x_2)^{j_2}g_3(x_2)^{j_3}}{M^{1-|J|}}\\&\qquad \times \frac{(0, g_3(x_2), -g_2'(x_2))}{g_3(x_2)^2 + g_2'(x_2)^2} + \frac{O_\delta (1)}{M^{2-|J|}}. \end{aligned}$$

We first integrate by parts again, absorbing back the constant $c_J$. If the derivative falls on $\chi$, integration by parts in $h_3$ shows that the integral in $h_3$ is $\lesssim _k M^{-k}(h_1^2 + h_2^2)^{-(1+k)/2} \le M^{1-k\delta } \le M^{-2}$ (taking $k = [3/\delta ] + 1$). Since the support of $\chi (M^{1-\delta }h)$ has size $O(M^{-2+2\delta })$ and its gradient has size $O(M^{1-\delta })$, the integral is under control. Since taking the derivative of the exponential means multiplication by $ig_2'(x_2)$ or $ig_3(x_2)$, it suffices to show the case when $J = 0$ and all factors involving *J* disappear. Then we undo the cross product with $e_1$, changing the vector on the right-hand side to $(0, g_2'(x_2), g_3(x_2))$. Next, using $h/|h|^3 = -\nabla (1/|h|)$, we can move one more derivative to the exponentials. Then we change variables and it suffices to show that

$$ \left| \int _{\mathbb {R}^3} \frac{\chi (h/M^\delta )e^{ig_2'(x_2)h_2}e^{ig_3(x_2)h_3}}{4\pi |h|}\text {d}h - \frac{1}{g_3(x_2)^2 + g_2'(x_2)^2} \right| \lesssim _\delta M^{-1} $$

where $\chi (h) = \chi (h_1, h_2)$ is a smooth bump function equal to 1 near the origin. Viewing $M^\delta$ as a whole, it suffices to show that, for $M \ge 1$ and $n \in \mathbb {N}$,

$$ \left| \int _{\mathbb {R}^3} \frac{\chi (h/M)e^{ig_2'(x_2)h_2}e^{ig_3(x_2)h_3}}{4\pi |h|}\text {d}h - \frac{1}{g_3(x_2)^2 + g_2'(x_2)^2} \right| \lesssim _n M^{-n}. $$

Let $\mathcal {F}_n$ be the Fourier transform in $\mathbb {R}^n$. Then it suffices to show that

$$ \left| \mathcal {F}_3\frac{\chi (h/M)}{4\pi |h|}(0, -g_2'(x_2), -g_3(x_2)) - \frac{1}{g_3(x_2)^2 + g_2'(x_2)^2} \right| \lesssim _n M^{-n}. $$

Since the Fourier transform takes products to convolutions,

$$ \mathcal {F}_3\frac{\chi (h/M)}{4\pi |h|} = \mathcal {F}_3(\chi (h/M)) * \mathcal {F}_3\frac{1}{4\pi |h|} = M^3\mathcal {F}_3\chi (M\xi ) * \frac{1}{|\xi |^2}. $$

Since $\chi$ is independent of $h_3$, $\mathcal {F}_3\chi = \delta (\xi _3)\mathcal {F}_2\chi$. Since $\int _{\mathbb {R}} \delta (M\xi _3)\text {d}\xi _3 = 1/M$,

$$ \mathcal {F}_3\frac{\chi (h/M)}{4\pi |h|}(\xi ) = M^2\int _{\mathbb {R}^2} \frac{\mathcal {F}_2\chi (M\eta )}{|\xi - \eta |^2}d{\eta }, $$

where $$\xi - \eta = (\xi _1 - \eta _1, \xi _2 - \eta _2, \xi _3)$$.

For $j + k > 0$, $\partial _1^j\partial _2^k\chi (0, 0) = 0$, so $\int _{\mathbb {R}^2} \mathcal {F}_2\chi (M\eta )\eta _1^j\eta _2^kd\eta = 0$, so

$$\begin{aligned}&\left| \int _{\mathbb {R}^2} \frac{\mathcal {F}_2\chi (M\eta )}{|\xi - \eta |^2}\text{ d }\eta - \frac{1}{|\xi |^2}\int _{\mathbb {R}^2} \mathcal {F}_2\chi (M\eta )\text{ d }\eta \right| \\  &\quad \lesssim _n \int _{|\xi -\eta |\ge |\xi |/2} |\mathcal {F}_2\chi (M\eta )|\frac{|\eta |^nd\eta }{|\xi |^{n+2}}\\  &\quad {(\text {when}\,\,\, |\eta |< |\xi |/2 \,\,\,\text {we} \,\,\,\text {use }\,\,\,\mathrm {Taylor's} \,\,\,\text {theorem;}\,\,\,\text {otherwise} \,\,\,\mathrm {it's}\,\,\,\text {trivial})}\\  &\qquad + \frac{1}{\xi _3^2}\int _{|\xi -\eta |<|\xi |/2} |\mathcal {F}_2\chi (M\eta )|\text{ d }\eta \quad {(|\xi |, |\xi - \eta | \ge |(\xi - \eta )_3| = |\xi _3|)}\\  &\lesssim _n \int _{\mathbb {R}^2} \frac{|\mathcal {F}_2\chi (\eta )||\eta |^n}{|M\xi |^{n+2}}\text{ d }\eta _1\text{ d }\eta _2 + \frac{1}{\xi _3^2|M\xi |^{n+2}}\int _{\mathbb {R}^2} \frac{\text{ d }\eta _1\text{ d }\eta _2}{(1 + |\eta |)^3}\\  &\quad \lesssim _n \frac{1 + \xi _3^{-2}}{|M\xi |^{n+2}}.\\  &\quad {(|\mathcal {F}_2\chi (M\eta )| \lesssim _n (1 + |M\eta |)^{-n-5}, M \ge 1\ and\ |\eta | \ge |\xi | - |\xi - \eta | > |\xi |/2)} \end{aligned}$$

Since $$\int _{\mathbb {R}^2} \mathcal {F}_2\chi (M\eta )\text {d}\eta = \chi (0)M^{-2} = M^{-2}$$,

$$ \left| \mathcal {F}_3\frac{\chi (h/M)}{4\pi |h|}(\xi ) - \frac{1}{|\xi |^2} \right| \lesssim _n \frac{1 + \xi _3^{-2}}{M^n|\xi |^{n+2}}, $$

giving the desired bound since $|\xi | \ge |\xi _3| = g_3(x_2) \ge 1/2$. $\square$

#### Corollary 1

For $$j = 0, 1$$,

$$\begin{aligned} \Vert \tilde{u}\Vert _{C^j}&\lesssim (M + B)^j/M,\\ \Vert \tilde{u} - \omega _1e_1 * \mathcal {K}\Vert _{C^j}&\lesssim _\delta BM^{(j-2)(1-\delta )}, \end{aligned}$$

because $|\tilde{u}_{,J}| \lesssim M^{|J|-1}$ and for $|J| = 1$, $|\partial _J\tilde{u} - \tilde{u}_{,J}| \lesssim B/M$.

Now we let $a(x_2) = 1/g_1(x_2)$ in the expression of $\omega _1$, so $\sup |a'| \lesssim \sup |g_1'|$. The respective bound has *B* replaced by $B_0:= B$ without the term $\sup |a'|$, i.e.,

$$ B_0 = \sum _{i=1}^3 L_i + L_3\ln M + \sup |g_1'| + ML_3^{-1}(\sup |g_3'| + \sup |g_2''|). $$

#### Lemma 6

For $$|J| \le 1$$,

$$ |\nabla \tilde{u}_{,J} - \partial _J\nabla (\omega _1e_1 * \mathcal {K})| \lesssim _\delta B_1M^{(|J|-2)(1-\delta )} $$

where $$B_1 = B_0(M + B_0) + \sup |g_1''| + ML_3^{-1}\sup |g_3''|$$.

#### Corollary 2

Similarly $$\Vert \tilde{u}_{,J}\Vert _{C^1} \lesssim M + B_0$$ and $$\Vert \omega _1e_1 * \mathcal {K}\Vert _{C^2} \lesssim _\delta M + B_1M^{-1+\delta }$$.

#### Proof of Lemma 6

When we take derivatives of

$$ g_1(x_2)^{-1}\psi (L_1g_1(x_2)x_1)\sin (Mg_2(x_2))\psi (L_2g_2(x_2))\sin (Mg_3(x_2)x_3)\psi (L_3g_3(x_2)x_3) $$

in various directions, they may fall on different factors. In addition to changing $\psi$ to $\psi '$ and sin to cos, which do not affect the form of the bound, it will also change $g_1(x_2)^{-1}$ to different factors $a(x_2)$, leading to different bounds, as detailed below:

**Table:** 

Factor hit by $\partial$	$a(x_2)$	$|a'(x_2)| \lesssim$	Effect on $B_0 \lesssim _\delta$
$\partial _1\psi (L_1g_1x_1)$	$L_1$	0	$B_0L_1 \le B_0^2$
$\partial _3\sin (Mg_3x_3)$	$Mg_3/g_1$	$M(\sup |g_1'| + \sup |g_3'|)$	$B_0M$
$\partial _3\psi (L_3g_3x_3)$	$L_3g_3/g_1$	$L_3(\sup |g_1'| + \sup |g_3'|)$	$B_0L_3 \le B_0^2$
$\partial _2(1/g_1(x_2))$	$-g_1'/g_1^2$	$\sup |g_1''| + \sup g_1'^2$	$B_0\sup |g_1'| + \sup |g_1''|$
$\partial _2\sin (Mg_2(x_2))$	$Mg_2'/g_1$	$M(\sup |g_1'| + \sup |g_2''|)$	$B_0M$
$\partial _2\psi (L_2g_2(x_2))$	$L_2g_2'/g_1$	$L_2(\sup |g_1'| + \sup |g_2''|)$	$B_0L_2 \le B_0^2$

Since

$$ \partial _2\psi (L_1g_1(x_2)x_1) = L_1g_1(x_2)x_1\psi '(L_1g_1(x_2)x_1)(g_1'/g_1)(x_2), $$

$|L_1x_1| \lesssim 1$ on the support of $\psi$ and $x\psi '(x)$ is smooth and compactly supported, we get an effect similar to that of $\partial _2(1/g_1(x_2))$. Similarly the effect of $\partial _2\psi (L_3g_3(x_2)x_3)$ on $B_0$ is $B_0\sup |g_3'| + \sup |g_3''|$. Also due to the support of $\psi (L_3g_3(x_2)x_3)$, the effect of $\partial _2\sin (Mg_3(x_2)x_3)$ is $O(M/L_3)$ times larger, so it is $\lesssim _\delta B_0ML_3^{-1}\sup |g_3'| + ML_3^{-1}\sup |g_3''| \le B_0^2 + ML_3^{-1}\sup |g_3''| \le B_1$. Finally in $\partial _2\tilde{u}_{,J}$ with $J \ne (1, 0, 0)$, there are extra terms coming from

$$ \left| \partial _2\frac{(g_2'(x_2)^{j_2}g_3(x_2)^{1+j_3}, g_2'(x_2)^{1+j_2}g_3(x_2)^{j_3})}{g_3(x_2)^2 + g_2'(x_2)^2} \right| \lesssim \sup |g_3'| + \sup |g_2''| \le B_0 $$

contributing $B_0M^{|J|-1} \le B_1M^{|J|-2} \le B_1M^{(|J|-2)(1-\delta )}$ to the bound. $\square$

### Quadratic Estimates

Now we add the other nonzero component to the vorticity. Let $\omega = (\omega _1, 0, \omega _3)$, where

11 $$\begin{aligned} \omega _3 = \int _{-\infty }^{x_3} \partial _1\omega _1(x_1, x_2, s)\text {d}s, \end{aligned}$$

so that $\omega$ is divergence-free. Note that since $\omega _1$ is odd in $x_3$, the support of $\omega _3$ is contained in that of $\omega _1$.

We first control the error in the quadratic self-interaction. The same heuristics yield a saving of order $\sum L_i/M$ compared to the trivial bound. Note that the factor $M + B_0$ in the censuring bound will turn out to be comparable to *M*.

#### Lemma 7

For $$s \in [0, 1]$$,

$$ \Vert (\omega * \mathcal {K}) \cdot \nabla \omega \Vert _{C^s} + \Vert \omega \cdot \nabla (\omega * \mathcal {K})\Vert _{C^s} \lesssim _\delta B_0^{1-s}B_1^sM^{-2+2\delta }(M + B_0). $$

#### Proof

Recall that

$$\begin{aligned} \omega _1&= -g_1(x_2)^{-1}\psi (L_1g_1(x_2)x_1)\sin (Mg_2(x_2))\psi (L_2g_2(x_2))\\&\quad \times \sin (Mg_3(x_2)x_3)\psi (L_3g_3(x_2)x_3). \end{aligned}$$

Each factor is *O*(1), and their higher derivative bounds are (constants omitted): $\square$

**Table:** 

Term	First derivative	Second derivative
$1/g_1(x_2)$	$\sup |g_1'|$	$\sup g_1'^2 + \sup |g_1''|$
$\psi (L_1g_1x_1)$	$L_1 + \sup |g_1'|$	$L_1^2 + \sup g_1'^2 + \sup |g_1''|$
$\sin (Mg_2)$	*M*	$M^2 + M\sup |g_2''|$
$\psi (L_2g_2)$	$L_2$	$L_2^2 + L_2\sup |g_2''|$
$\psi (L_3g_3x_3)$	$L_3 + \sup |g_3'|$	$L_3^2 + \sup g_3'^2 + \sup |g_3''|$
$\cos (Mg_3x_3)$	$M(1 + \sup |g_3'|/L_3)$	$M^2(1 + \sup g_3'^2/L_3^2) + ML_3^{-1}\sup |g_3''|$
$\omega _1$	$M + B_0$	$M^2 + B_1$

Similarly, the corresponding bounds for $\tilde{u}$ are smaller by a factor of *M*.

Note that $\partial _2\omega _1 - Mg_2'(x_2)\omega _1\cot (Mg_2(x_2))$ is a sum of functions like $\omega _1$ with the following factor replacements, with corresponding derivative bounds:

**Table:** 

Factor	Replaced by	sup	First derivative
$1/g_1$	$-g_1'/g_1^2$	$\sup |g_1'|$	$\sup g_1'^2 + \sup |g_1''|$
$\psi (L_1g_1x_1)$	$L_1g_1'\psi '(L_1g_1x_1)x_1$	$\sup |g_1'|$	$L_1\sup |g_1'| + \sup g_1'^2 + \sup |g_1''|$
$\psi (L_2g_2)$	$L_2g_2'\psi '(L_2g_2)$	$L_2$	$L_2^2 + L_2\sup |g_2''|$
$\psi (L_3g_3x_3)$	$L_3g_3'\psi '(L_3g_3x_3)x_3$	$\sup |g_3'|$	$L_3\sup |g_3'| + \sup g_3'^2 + \sup |g_3''|$
$\sin (Mg_3x_3)$	$Mg_3'\cos (Mg_3x_3)x_3$	$ML_3^{-1}\sup |g_3'|$	$* \le B_1$

where $* = M^2L_3^{-1}\sup |g_3'| + M^2L_3^{-2}\sup g_3'^2 + ML_3^{-1}\sup |g_3''|$. Then for $j = 0, 1$, using $\Vert \tilde{u}\Vert _{C^j} \lesssim (M + B)^j/M$ and $B_0(M + B_0) \le B_1$ we have that

$$\begin{aligned} \Vert \partial _2\omega _1 - Mg_2'(x_2)\omega _1\cot (Mg_2(x_2))\Vert _{C^j}&\lesssim B_j,\\ \Vert \tilde{u}_2\partial _2\omega _1 - \tilde{u}_2Mg_2'(x_2)\omega _1\cot (Mg_2(x_2))\Vert _{C^j}&\lesssim B_j/M. \end{aligned}$$

Also, $\partial _3\omega _1 - Mg_3(x_2)\omega _1\cot (Mg_3(x_2)x_3)$ is $\omega _1$ with the following factor replaced, with corresponding bounds on $C^0$ and $C^1$ norms:

**Table:** 

Factor	Replaced by	sup	First derivative
$\psi (L_3g_3x_3)$	$L_3g_3\psi '(L_3g_3x_3)$	$L_3$	$L_3^2 + L_3\sup |g_3'|$

Then for $j = 0, 1$, using $L_3 + \sup |g_3'| \le B_0$ and $\Vert \tilde{u}\Vert _{C^j} \lesssim (M + B_0)^j/M$ we have that

$$\begin{aligned} \Vert \partial _3\omega _1 - Mg_3(x_2)\omega _1\cot (Mg_3(x_2)x_3)\Vert _{C^j}&\lesssim L_3(M + B_0)^j,\\ \Vert \tilde{u}_3\partial _3\omega _1 - \tilde{u}_3Mg_3(x_2)\omega _1\cot (Mg_3(x_2)x_3)\Vert _{C^j}&\lesssim L_3(M + B_0)^j/M. \end{aligned}$$

Since $L_3 \le B_0$ and *M*, $L_3(M + B_0) \le 2MB_0 \le 2B_1$, so the first bound dominates the second one. Since $\tilde{u}_2g_2'(x_2)\cot (Mg_2(x_2)) + \tilde{u}_3g_3(x_2)\cot (Mg_3(x_2)x_3) = 0$,

$$ \Vert \tilde{u}\cdot \nabla \omega _1\Vert _{C^j} \lesssim B_j/M. $$

Integrating by parts we have

$$\begin{aligned} \omega _3&= -L_1\psi '(L_1g_1(x_2)x_1)\sin (Mg_2(x_2))\psi (L_2g_2(x_2))I,\\ I&= \int _{-\infty }^{x_3} \sin (Mg_3(x_2)y)\psi (L_3g_3(x_2)y)\text {d}y = I_0 + R_0,\\ I_0&= -\frac{\cos (Mg_3(x_2)x_3)\psi (L_3g_3(x_2)x_3)}{Mg_3(x_2)},\\ R_0&= \int _{-\infty }^{x_3} \frac{\cos (Mg_3(x_2)y)L_3\psi '(L_3g_3(x_2)y)}{M}\text {d}y\\&= \int _{-\infty }^{L_3x_3} \frac{\cos (ML_3^{-1}g_3(x_2)y)\psi '(g_3(x_2)y)}{M}\text {d}y. \end{aligned}$$

The derivative bounds are

**Table:** 

Term	sup	First derivative	Second derivative
$\psi (L_3g_3x_3)$	1	$L_3 + \sup |g_3'|$	$L_3^2 + \sup g_3'^2 + \sup |g_3''|$
$\cos (Mg_3x_3)$	1	$M(1 + \sup |g_3'|/L_3)$	$M^2(1 + \sup g_3'^2/L_3^2) + ML_3^{-1}\sup |g_3''|$
$1/g_3(x_2)$	1	$\sup |g_3'|$	$\sup g_3'^2 + \sup |g_3''|$
$I_0$	1/*M*	$1 + \sup |g_3'|/L_3$	$M(1 + \sup g_3'^2/L_3^2) + \sup |g_3''|/L_3$
$R_0$	1/*M*	$\frac{1 + \sup |g_3'|}{L_3} + L_3/M$	$\frac{M^2(1 + \sup g_3'^2)}{L_3^2} + \sup |g_3''|/L_3 + L_3 + \sup |g_3'|$
*I*	1/*M*	$1 + \sup |g_3'|/L_3$	$M(1 + \sup g_3'^2/L_3^2) + \sup |g_3''|/L_3$

where we have used $L_3 + \sup |g_3'| \le M(1 + \sup |g_3'|/L_3) \lesssim M(1 + \sup g_3'^2/L_3^2)$ to dominate $R_0$ with $I_0$. Continuing,

**Table:** 

Term	sup	First derivative	Second derivative
$\psi '(L_1g_1x_1)$	1	$L_1 + \sup |g_1'|$	$L_1^2 + \sup g_1'^2 + \sup |g_1''|$
$\sin (Mg_2)$	1	*M*	$M^2 + M\sup |g_2''|$
$\psi (L_2g_2)$	1	$L_2$	$L_2^2 + L_2\sup |g_2''|$
*I*	1/*M*	$1 + \sup |g_3'|/L_3$	$M(1 + \sup g_3'^2/L_3^2) + \sup |g_3''|/L_3$
$M\omega _3/L_1$	1	$M + B_0$	$M^2 + B_1$

Then for $j = 0, 1$, using $\Vert \tilde{u}\Vert _{C^j} \lesssim (M + B)^j/M$ and $(M + B_0)^2 \le M^2 + B_1$ we have that

$$\begin{aligned} \Vert \nabla \omega _3\Vert _{C^j}&\lesssim L_1(M^{j+1} + B_j)/M,\\ \Vert \tilde{u}\cdot \nabla \omega _3\Vert _{C^j}&\lesssim L_1(M^{j+1} + B_j)/M^2. \end{aligned}$$

Since $L_1M^j \le B_j$ and $L_1 \le M$, $L_1(M^{j+1} + B_j) \le B_j(L_1 + M) \le 2B_jM$, so

$$ \Vert \tilde{u}\cdot \nabla \omega _3\Vert _{C^j} \lesssim B_j/M,\quad \Vert \tilde{u}\cdot \nabla \omega \Vert _{C^j} \lesssim B_j/M. $$

Since $$\Vert \tilde{u} - \omega _1e_1 * \mathcal {K}\Vert _{C^j} \lesssim _\delta B_0M^{(j-2)(1-\delta )}$$, $$\Vert \nabla \omega \Vert _{C^j} \lesssim M^{j+1} + B_j$$ and $$M(M + B_0) \le M^2 + B_1$$,

$$ \Vert (\tilde{u} - \omega _1e_1 * \mathcal {K})\cdot \nabla \omega \Vert _{C^j} \lesssim _\delta B_0M^{-2+2\delta }(M^{j+1} + B_j), $$

so using $$B_0M \le B_1$$, $$\Vert (\omega _1e_1 * \mathcal {K})\cdot \nabla \omega \Vert _{C^j} \le B_jM^{-2+2\delta }(M + B_0)$$.

Turning to

$$\begin{aligned} \omega _3&= -L_1\psi '(L_1g_1(x_2)x_1)\sin (Mg_2(x_2))\psi (L_2g_2(x_2))I,\\ I&= \int _{-\infty }^{x_3} \sin (Mg_3(x_2)s)\psi (L_3g_3(x_2)y)\text {d}y = \sum _{i=0}^k I_i + R_k,\\ I_i&= -\frac{\cos (Mg_3(x_2)x_3 + i\pi /2)L_3^i\psi ^{(i)}(L_3g_3(x_2)x_3)}{M^{i+1}g_3(x_2)},\\ R_k&= \int _{-\infty }^{L_3x_3} \frac{\cos (ML_3^{-1}g_3(x_2)y + k\pi /2)L_3^k\psi ^{(k+1)}(g_3(x_2)y)}{M^{k+1}}\text {d}y, \end{aligned}$$

the term involving $I_i$ has the same form as $\omega _1$, with

$$ a(x_2) = L_1L_3^i/(M^{i+1}g_3(x_2)), $$

so its convolution with the Biot–Savart kernel has an estimate whose main term and error term are both $L_1L_3^i/M^{i+1}$ times that of $\omega _1$. Then for $j = 0, 1$,

$$ \Vert (\text {part of }{\omega _3}\text { involving }{I_i})e_3 * \mathcal {K}\Vert _{C^j} \lesssim _\delta L_1L_3^i(M + B_0)M^{(j-2)(1-\delta )-i-1}. $$

Let $$k = [3/\delta ] + 1$$. Since $$L_i \le M$$ ($$i = 1, 2, 3$$),

$$ \Vert \text{ part } \text{ of } {\omega _3} \text{ involving } { R_k}\Vert _{C^j} \lesssim L_1L_3^kM^{j-k-1} \lesssim _\delta L_1/M^{3}, $$

so, since supp $\omega _3 \subset \{|x| \le 1\}$ and the Biot–Savart kernel is locally integrable,

$$ \Vert (\text {part of }{\omega _3} \text { involving }{R_k})e_3 * \mathcal {K}\Vert _{C^j} \lesssim _\delta L_1M^{-3}. $$

Since $M \ge 1$, summing over $I_0, \dots , I_k$ and $R_k$ we get, for $j = 0, 1$,

$$ \Vert \omega _3e_3 * \mathcal {K}\Vert _{C^j} \lesssim _\delta L_1(M + B_0)M^{(j-2)(1-\delta )-1}. $$

Since $$\Vert \nabla \omega \Vert _{C^j} \lesssim M^{j+1} + B_j$$, $$M(M + B_0) \le M^2 + B_1$$ and $$L_1(M^{j+1} + B_j) \le B_j(L_1 + M) \le 2B_jM$$,

$$ \Vert (\omega _3e_3 * \mathcal {K})\cdot \nabla \omega \Vert _{C^j} \lesssim _\delta L_1(M^{j+1} + B_j)M^{-3+2\delta }(M + B_0) \le B_jM^{-2+2\delta }(M + B_0), $$

so $(\omega * \mathcal {K})\cdot \nabla \omega$ also has the same bound.

Turning to the other term, we have

$$ \Vert \partial _1\tilde{u}\Vert _{C^j} \lesssim L_1M^{j-1}. \quad {(j = 0, 1)}, $$

Since $\partial _1\omega _1$ is of the same form as $\omega _1$, with the factor $1/g_1$ replaced by $L_1$,

$$ \Vert \partial _1(\tilde{u} - \omega _1e_1 * \mathcal {K})\Vert _{C^j} \lesssim _\delta L_1B_0M^{(j-2)(1-\delta )} \quad {(j = 0, 1)} $$

so

$$ \Vert \partial _1(\omega _1e_1 * \mathcal {K})\Vert _{C^j} \lesssim _\delta L_1(M + B_0)M^{(j-2)(1-\delta )}. \quad {(j = 0, 1)} $$

Since $$\Vert \omega _1\Vert _{C^j} \lesssim (M + B_0)^j$$ ($$j = 0, 1$$),

$$ \Vert \omega _1\partial _1(\omega _1e_1 * \mathcal {K})\Vert _{C^j} \lesssim _\delta L_1(M + B_0)^{j+1}M^{-2+2\delta }. \quad {(j = 0, 1)} $$

Since $\partial _3\omega _1$ is of the same form as $\omega _1$, with the factor $1/g_1$ replaced by $Mg_3/g_1$ and $L_3g_3/g_1$, the bounds for $\partial _3(\omega _1e_1 * \mathcal {K})$ are $M/L_1$ times those of $\partial _1(\omega _1e_1 * \mathcal {K})$. Since the bounds for $\omega _3$ are $L_1/M$ times those of $\omega _1$, $\omega _3\partial _3(\omega _1e_1 * \mathcal {K})$ has the same bounds as $\omega _1\partial _1(\omega _1e_1 * \mathcal {K})$, so $\omega \cdot \nabla (\omega _1e_1 * \mathcal {K})$ also has the same bounds.

Since $\partial _1\omega _1$ is of the same form as $\omega _1$, with the factor $1/g_1$ replaced by $L_1$, for $j = 0, 1$,

$$ \Vert \partial _1(\omega _3e_3 * \mathcal {K})\Vert _{C^j} \lesssim _\delta L_1^2(M + B_0)M^{(j-2)(1-\delta )-1}. $$

Since $$\Vert \omega _1\Vert _{C^j} \lesssim (M + B_0)^j$$,

$$ \Vert \omega _1\partial _1(\omega _3e_3 * \mathcal {K})\Vert _{C^j} \lesssim _\delta L_1^2(M + B_0)^{j+1}M^{-3+2\delta }. $$

Similarly $\omega _3\partial _3\nabla (\omega _3e_3 * \mathcal {K})$ also has the same bounds. Since $L_1(M + B_0)^j \le M(M + B_0)^j \le B_1$,

$$ \Vert \omega \cdot \nabla (\omega * \mathcal {K})\Vert _{C^j} \lesssim _\delta L_1(M + B_0)^{j+1}M^{-2+2\delta } \le B_j(M + B_0)M^{-2+2\delta }. \quad {(j = 0, 1)} $$

The results now follow from interpolation and $B_0 \le MB_0 \le B_1$. $\square$

#### Lemma 8

Assume that $g_2(0) = 0$. Then

$$\begin{aligned} \left| \partial _3(\omega * \mathcal {K})_3(0, 0, 0) + \frac{g_2'(0)g_3(0)}{g_1(0)(g_3(0)^2 + g_2'(0)^2)} \right|&\lesssim _\delta B_0M^{\delta -1},\\ |\nabla (\omega * \mathcal {K})_1|&\lesssim _\delta L_1(M + B_0)M^{\delta -2}. \end{aligned}$$

#### Proof

In the proof of Lemma 7 we saw $\Vert \omega _3e_3 * \mathcal {K}\Vert _{C^1} \lesssim _\delta L_1(M + B_0)M^{\delta -2}$. Since $(\omega _1e_1 * \mathcal {K})_1 = 0$, we get the second bound. The second term in the first bound is exactly $-\partial _3\tilde{u}_3(0, 0, 0)$, so by combining Lemma 5 and the $C^1$ bound for $\omega _3e_3 * \mathcal {K}$, we get the first bound, which dominates the second one because $L_1M$ and $L_1B_0 \le B_0M$. $\square$

Then we control the error in the bilinear interaction between the outer and inner vortex layers. Let $D = \max \{|x|: x \in \text {supp }\omega \}$. It measures how large $\omega$ expands, and depends on $L_i$ and $g_i$ ($i = 1, 2, 3$). Far away from the support of $\omega$, the oscillation of $\omega$ makes the velocity field decay very rapidly.

#### Lemma 9

There is an absolute constant *C* such that if $|x| > CD$, then for all $j, n = 0, 1, \dots$, $|\nabla ^j(\omega * \mathcal {K})(x)| \lesssim _{j,n,\delta } M^{-n}$.

#### Proof

We first show the result for $j = 0$. Recall that

$$ \omega _1e_1 * \mathcal {K} = \int _{\mathbb {R}^3} \frac{h\times e_1}{4\pi |h|^3}\omega _1(x + h)\text {d}h. $$

In the proof of Lemma 5 we have shown, for any $k \in \mathbb {N}$,

$$\begin{aligned}&\left| \int _{\mathbb {R}} \frac{h\times e_1\sin (Mg_3(x_2 + h_2)(x_3 + h_3))\psi (L_3g_3(x_2 + h_2)(x_3 + h_3))}{|h|^3}\text {d}h_3 \right| \\&\lesssim _k M^{-k}\sum _{j=0}^k L_3^j(h_1^2 + h_2^2)^{-(1+k-j)/2}. \end{aligned}$$

Since $D \gtrsim 1/L_1 \ge M^{-1+\delta }$, if $|x_1|$ or $|x_2| > CD$ then on the support of $\omega _1$, $h_1^2 + h_2^2 \ge M^{-2+2\delta }$, so we get the desired bound as in the proof of Lemma 5. If $|x_3| > CD$, then similarly on the support of $\omega _1$, $|h_3| \ge M^{-1+\delta }$, so when integrating by parts in $h_3$ we get

$$\begin{aligned}&\left| \int _{\mathbb {R}} \frac{h\times e_1\sin (Mg_3(x_2 + h_2)(x_3 + h_3))\psi (L_3g_3(x_2 + h_2)(x_3 + h_3))}{|h|^3}\text {d}h_3 \right| \\&\lesssim _k M^{-k}\sum _{j=0}^k L_3^j\int _{M^{-1+\delta }}^\infty \frac{|h_1| + |h_2| + h_3}{|h|^{3+k-j}}\text {d}h_3 \le M^{-k}\sum _{j=0}^k L_3^j\int _{M^{-1+\delta }}^\infty \frac{3\text {d}h_3}{|h|^{2+k-j}}\\&\lesssim _k M^{1-k}\sum _{j=0}^k L_3^jM^{(1-\delta )(k-j)} \lesssim _k M^{1-\delta k} \lesssim _{n,\delta } M^{-n}. \quad {(k := [\frac{n+1}{\delta }] + 1)} \end{aligned}$$

Now we integrate in $h_1$ and $h_2$, noting that the area of the domain of integration is $\pi$.

Turning to

$$\begin{aligned} \omega _3&= -L_1\psi '(L_1g_1(x_2)x_1)\sin (Mg_2(x_2))\psi (L_2g_2(x_2))I,\\ I&= \int _{-\infty }^{x_3} \sin (Mg_3(x_2)s)\psi (L_3g_3(x_2)y)\text {d}y = \sum _{i=0}^k I_i + R_k,\\ I_i&= -\frac{\cos (Mg_3(x_2)x_3 + i\pi /2)L_3^i\psi ^{(i)}(L_3g_3(x_2)x_3)}{M^{i+1}g_3(x_2)},\\ R_k&= \int _{-\infty }^{L_3x_3} \frac{\cos (ML_3^{-1}g_3(x_2)y + k\pi /2)L_3^k\psi ^{(k+1)}(g_3(x_2)y)}{M^{k+1}}\text {d}y. \end{aligned}$$

Let $$k = [n/\delta ] + 1$$. Since $$L_3 \le M^{1-\delta }$$,

$$ \Vert \text {part of}{ \omega _3} \text { involving }{R_k}\Vert _{C^0} \lesssim _k L_1L_3^kM^{-k-1} \lesssim _{n,\delta } M^{-n}, $$

so, since supp $\omega _3 \subset \{|x| \le 1\}$ and the Biot–Savart kernel is locally integrable,

$$ \Vert (\text {part of }{\omega _3} \text { involving }{R_k})e_3 * \mathcal {K}\Vert _{C^0} \lesssim _{n,\delta } M^{-n}. $$

Since the terms involving $I_i$ has similar estimates as $L_1L_3^iM^{-i-1}\omega _1$, and $L_1, L_3 \le M$, their convolutions with the Biot–Savart kernel has the same bound as $\omega _1e_1 * \mathcal {K}$, so does $\omega _3e_3 * \mathcal {K}$, and hence so does $\omega * \mathcal {K}$.

For $j > 0$, we fall the derivatives on the Biot–Savart kernel. Since $|h| \ge M^{1-\delta }$, the bound is larger by a factor of $M^{j(1-\delta )}$, so the result still holds. $\square$

#### Lemma 10

Let $\Omega$ be an axial vector field. Then

$$\begin{aligned} \Vert (\omega * \mathcal {K}) \cdot \nabla \Omega \Vert _{C^j}&\lesssim _\delta (M + B_0)\sum _{j_1+j_2=j}M^{(j_1-2)(1-\delta )}\Vert \Omega \Vert _{C^{j_2+1}}, \quad {(j = 0, 1)}\\ \Vert \Omega \cdot \nabla (\omega * \mathcal {K})\Vert _{C^s}&\lesssim _\delta (M + B_0)M^{(s-1)(1-\delta )}D\Vert \Omega \Vert _{C^1}. \quad {(s \in [0, 1])} \end{aligned}$$

#### Proof

The first bound follows from $\Vert \omega * \mathcal {K}\Vert _{C^j} \lesssim _\delta (M + B_0)M^{(j-2)(1-\delta )}$ ($j = 0, 1$).

For the second bound, when $|x_i| > CD$ for some $i = 1, 2, 3$, by Lemma 9, $\Vert \nabla (\omega * \mathcal {K})\Vert _{C^1} \lesssim _\delta M^{-1}$ so $\Vert \Omega \cdot \nabla (\omega * \mathcal {K})\Vert _{C^j} \lesssim _\delta M^{-1}\Vert \Omega \Vert _{C^j}$ for $j = 0, 1$. When $|x_i| \le CD$ for all $i = 1, 2, 3$, since $\Omega$ is odd, $\Omega (0) = 0$, so $\Vert \Omega \Vert _{C^j} \lesssim D^{1-j}\Vert \Omega \Vert _{C^1}$ for $j = 0, 1$. Since $\Vert \nabla (\omega * \mathcal {K})\Vert _{C^j} \lesssim _\delta (M + B_0)M^{(j-1)(1-\delta )}$,

$$\begin{aligned} \Vert \Omega \cdot \nabla (\omega * \mathcal {K})\Vert _{C^j}&\lesssim _\delta \sum _{j_1+j_2=j} (M + B_0)M^{(j_1-1)(1-\delta )}D^{1-j_2}\Vert \Omega \Vert _{C^1}\\&\lesssim _\delta (M + B_0)M^{(j-1)(1-\delta )}D\Vert \Omega \Vert _{C^1}. \quad {(j = 0, 1)} \end{aligned}$$

because $D \gtrsim M^{-1}$. This dominates the previous bound $\Vert \Omega \cdot \nabla (\omega * \mathcal {K})\Vert _{C^j} \lesssim _\delta M^{-1}\Vert \Omega \Vert _{C^j}$. The result follows from interpolation. $\square$

#### Lemma 11

Let $\Omega$ be an axial vector field. Let

$$ U(x_1,x_2,x_3) = (x_1\partial _1(\Omega * \mathcal {K})_1(0, x_2, 0), (\Omega * \mathcal {K})_2(0, x_2, 0), x_3\partial _3(\Omega * \mathcal {K})_3(0, x_2, 0)). $$

Then for $$j = 0, 1$$,

$$\begin{aligned} \Vert (\Omega * \mathcal {K} - U) \cdot \nabla \omega \Vert _{C^j}&\lesssim _\delta (M^{j+1} + B_j)(1/L_1 + 1/L_3)^2D^{1-\delta }\Vert \Omega \Vert _{C^2},\\ \Vert \omega \cdot \nabla (\Omega * \mathcal {K} - U)\Vert _{C^j}&\lesssim _\delta (M + B_0)^j(1/L_1 + 1/L_3)D^{1-\delta }\Vert \Omega \Vert _{C^2}. \end{aligned}$$

#### Proof

Since $\Omega$ is axial, for $i = 1, 3$, $(\Omega * \mathcal {K} - U)_i(\cdot , x_2, \cdot )$ vanishes to order 3 at (0, 0). Then for $\beta = 1 - \delta \in (0, 1)$,

$$ |\nabla ^2(\Omega * \mathcal {K} - U)_i(x_1, x_2, x_3)| \le (|x_1|^\beta + |x_3|^\beta )\Vert \Omega * \mathcal {K}\Vert _{C^{2+\beta }} \lesssim _\beta (|x_1|^\beta + |x_3|^\beta )\Vert \Omega \Vert _{C^2} $$

so by Taylor’s theorem with the Lagrange remainder, for $j = 0, 1, 2$,

$$ |\nabla ^j(\Omega * \mathcal {K} - U)_i(x_1, x_2, x_3)| \lesssim _\delta (|x_1|^{3-j+\delta } + |x_3|^{3-j+\delta })\Vert \Omega \Vert _{C^2}. $$

For the second component, since $\Omega$ is odd, both $(\Omega * \mathcal {K})_2$ and $U_2$ vanish when $x_2 = 0$, so does $\nabla _{1,3}^2(\Omega * \mathcal {K} - U)_2$, so

$$ |\nabla _{1,3}^2(\Omega * \mathcal {K} - U)_2(x_1, x_2, x_3)| \le |x_2|^{1-\delta }\Vert \Omega * \mathcal {K}\Vert _{C^{3-\delta }} \lesssim _\delta |x_2|^{1-\delta }\Vert \Omega \Vert _{C^2}. $$

Since $(\Omega * \mathcal {K} - U)_2(\cdot , x_2, \cdot )$ vanishes to order 2 at (0, 0), for $j = 0, 1, 2$,

$$ |\nabla ^j(\Omega * \mathcal {K} - U)_2(x_1, x_2, x_3)| \lesssim _\delta (|x_1|^{2-j} + |x_3|^{2-j})|x_2|^{1-\delta }\Vert \Omega \Vert _{C^2}. $$

Since, on the support of $\omega$, $|x_i| \lesssim 1/L_i$ ($i = 1, 3$) and $|x_2| \le D$,

$$ \Vert \Omega * \mathcal {K} - U\Vert _{C^j({{\,\textrm{supp}\,}}\omega )} \lesssim _\delta (1/L_1 + 1/L_3)^{2-j}D^{1-\delta }\Vert \Omega \Vert _{C^2}. $$

Since $\Vert \omega \Vert _{C^j} \lesssim (M + B_0)^j$, $\Vert \nabla \omega \Vert _{C^j} \lesssim M^{j+1} + B_j$ ($j = 0, 1$) and $L_1, L_3 \le M$, the result follows. $\square$

Now we estimate the dissipation and control the error.

#### Lemma 12

For $$\alpha \in (0, 1)$$,

$$ ||\nabla |^\alpha \omega _1 - M^\alpha (g_2'(x_2)^2 + g_3(x_2)^2)^{\alpha /2}\omega _1| \lesssim _{\alpha ,\delta } BM^{(\alpha -1)(1-\delta )}, $$

where $$B = \sum _{i=1}^3L_i + L_3\ln M + \sup |a'| + \sup |g_1'| + ML_3^{-1}(\sup |g_3'| + \sup |g_2''|)$$.

#### Proof

Again we can replace all trignometric factors with exponential ones. We have

$$ |\nabla |^\alpha \omega _1 = c_\alpha \int \frac{\omega _1(x + h) - \omega _1(x)}{|h|^{3+\alpha }}\text {d}h,\quad c_\alpha = \frac{2^\alpha \Gamma (\frac{\alpha +3}{2})}{\pi ^{3/2}\Gamma (-\alpha /2)}. $$

Let $\chi (h) = \chi (h_1, h_2)$ is a smooth bump function equal to 1 near the origin. As in Lemma 5 we integrate by parts in $h_3$ to get

$$\begin{aligned}&\left| \int _{\mathbb {R}} \frac{e^{iMg_3(x_2 + h_2)(x_3 + h_3)}\psi (L_3g_3(x_2 + h_2)(x_3 + h_3))}{|h|^{3+\alpha }}\text {d}h_3 \right| \\&\lesssim _k M^{-k}\sum _{j=0}^k L_3^j(h_1^2 + h_2^2)^{-(2+\alpha +k-j)/2}, \end{aligned}$$

which is the same as the corresponding bound in Lemma 5 with $|J| = 1 + \alpha$, so, by the same reasoning,

$$ \int _{\mathbb {R}^2} (1 - \chi (M^{1-\delta }h))\int _{\mathbb {R}} \frac{\omega _1(x + h)}{|h|^{3+\alpha }}\text {d}h_3\text {d}h_1\text {d}h_2 $$

has the desired bound. Since $|h|^{-3-\alpha }$ is integrable at infinity,

$$\begin{aligned}&\int _{\mathbb {R}^2} (1 - \chi (M^{1-\delta }h))\int _{\mathbb {R}} \frac{a(x_2)\psi (L_1g_1(x_2)x_1)\psi (L_2g_2(x_2))\psi (L_3g_3(x_2)x_3)}{|h|^{3+\alpha }}\\&\times e^{iM(g_2(x_2)+g_2'(x_2)h_2+g_3(x_2)(x_3+h_3))}\text {d}h_3\text {d}h_1\text {d}h_2 \end{aligned}$$

also has the same bound, so in $(1 - \chi (M^{1-\delta }h))(\omega _1(x + h) - \omega _1(x))$, we can replace $\omega _1(x + h)$ with the planar wave

$$ a(x_2)\psi (L_1g_1(x_2)x_1)\psi (L_2g_2(x_2))\psi (L_3g_3(x_2)x_3)e^{iM(g_2(x_2)+g_2'(x_2)h_2+g_3(x_2)(x_3+h_3))}. $$

In $\chi (M^{1-\delta }h)(\omega _1(x + h) - \omega _1(x))$, we can also do so as in Lemma 5, with |*J*| replaced by $1 + \alpha$, with acceptable error. Now we end up with the action of $|\nabla |^\alpha$ on a factor independent of *h* times the planar wave $e^{iM(g_2'(x_2)h_2+g_3(x_2)h_3)}$, whose result is multiplication by $M^\alpha (g_2'(x_2)^2 + g_3(x_2)^2)^{\alpha /2}$; hence the result follows. $\square$

#### Corollary 3

If $\alpha \in (0, 1)$, $|g_2'| \le 1$ and $|g_3| \le 2$ then

$$\begin{aligned} ||\nabla |^\alpha \omega _1 - M^\alpha (g_2'(0)^2 + g_3(0)^2)^{\alpha /2}\omega _1|&\lesssim _{\alpha ,\delta } BM^{(\alpha -1)(1-\delta )}\\&+ M^\alpha |x_2|\sup (|g_2''| + |g_3'|), \end{aligned}$$

where *B* is the same as before.

#### Proof

Since $$|g_2'| \le 1$$ and $$|g_3| \le 2$$, $$(g_2'^2 + g_3^2)^{\alpha /2}|_0^{x_{n+2}} \lesssim |x_2|\sup (|g_2''| + |g_3'|)$$. $$\square $$

#### Corollary 4

If $\alpha \in (0, 1)$, $|g_2'| \le 1$, $|g_3| \le 2$ and $0 \le s < 1 - \alpha$ then

$$\begin{aligned} \Vert |\nabla |^\alpha \omega _1 - M^\alpha (g_2'(0)^2 + g_3(0)^2)^{\alpha /2}\omega _1\Vert _{C^s}&\lesssim _{s,\alpha ,\delta } BM^{\alpha +(s-1)(1-\delta )}\\&+ M^{\alpha +s}\sup _{{{\,\textrm{supp}\,}}\omega _1}|x_2|\sup (|g_2''| + |g_3'|), \end{aligned}$$

where *B* is the same as before.

#### Proof

Thanks to the embedding $L^\infty \cap |\nabla |^{-s}L^\infty \subset B^s_{\infty ,\infty } = C^s$, we only need to show the special case $s = 0$, and bound the following:

$$\begin{aligned}&||\nabla |^{\alpha +s}\omega _1 - M^\alpha (g_2'(0)^2 + g_3(0)^2)^{\alpha /2}|\nabla |^s\omega _1|\\&\le ||\nabla |^{\alpha +s}\omega _1 - M^{\alpha +s}(g_2'(0)^2 + g_3(0)^2)^{(\alpha +s)/2}\omega _1|\\&+ M^\alpha (g_2'(0)^2 + g_3(0)^2)^{\alpha /2} ||\nabla |^s\omega _1 - M^s(g_2'(0)^2 + g_3(0)^2)^{s/2}\omega _1|\\&\lesssim _{s,\alpha ,\delta } BM^{(\alpha +s-1)(1-\delta )} + BM^{\alpha +(s-1)(1-\delta )} + M^{\alpha +s}|x_2|\sup (|g_2''| + |g_3'|). \end{aligned}$$

Here the second term on the right-hand side dominates the first. This (together with the corresponding bound when $s = 0$) gives the desired bound when $|x_2| \le C\sup _{{{\,\textrm{supp}\,}}\omega _1}|x_2|$. Otherwise $\omega _1$ vanishes, and $||\nabla |^\alpha \omega _1|$, $||\nabla |^s\omega _1|$ and $||\nabla |^{\alpha +s}\omega _1| \lesssim _{\alpha ,s,\delta } M^{-1}$ by integration by parts in $x_2$ as in the proof of Lemma 9, and the bound also holds because $B \ge L_1 \ge 1$. $\square$

## The Construction

Let $\alpha \in (0, \frac{22-8\sqrt{7}}{9})$. Since $8\sqrt{7} = \sqrt{448} > \sqrt{441} = 21$, $\alpha < 1/9$. Let $R = \sqrt{\frac{2}{7\alpha }}$ and $s = (3\alpha +2-2\sqrt{14\alpha })/4$. Then $R > \sqrt{2}$ and $0< s< (\sqrt{3\alpha } + 2 - 2\sqrt{12\alpha })/4 < 1/2$.

Let $A = N^{\sqrt{2\alpha /7}}$ and $L = N^{1+\alpha -s-2\sqrt{2\alpha /7}}$. Then $L = N^{1+\frac{\alpha -2}{4}+\frac{3}{2}\sqrt{2\alpha /7}}< N^{1-\frac{17}{36}+\frac{3}{2}\sqrt{2/63}} < N^{5/6}$ and $L > N^{\frac{3}{2}\sqrt{2\alpha /7}} = A^{3/2}$. For $t \in [0, 1]$ let

$$ \omega _0(x, t) = A\psi (Lx_1)\psi (Lx_2)\psi (Lx_3)\sin (Nx_2)\sin (Nx_3)e_1 + \cdots e_{3}, $$

where the coefficient of $e_3$ is chosen to make $\omega _0$ divergence free, see (11). Suppose $\omega _k(x, t)$ is defined for $0 \le k \le n - 1$ and $t \in [0, 1]$. Let $\Omega _n = \sum _{k=0}^{n-1} \omega _k$ and

12 $$\begin{aligned} \begin{aligned} U_n(x, t)&= x_{n+1}\partial _{n+1}(\Omega _n * \mathcal {K})_{n+1}(x_{n+2}e_{n+2}, t)e_{n+1}\\&+ (\Omega _n * \mathcal {K})_{n+2}(x_{n+2}e_{n+2}, t)e_{n+2}\\&+ x_{n+3}\partial _{n+3}(\Omega _n * \mathcal {K})_{n+3}(x_{n+2}e_{n+2}, t)e_{n+3}, \end{aligned} \end{aligned}$$

where the subscripts on the right-hand side are taken modulo 3. Let $\phi _n$ be the flow of $U_n$. We then define $\omega _n$ on $[T_n, 1]$ (where $T_n$ is chosen later in (17)) by solving the transport equation

$$ \partial _t\omega _n + U_n\cdot \nabla \omega _n = \omega _n\cdot \nabla U_n - g_n(t)\omega _{n}, $$

where

$$ g_n(t) = \nu N^{\alpha R^n}(g_{n2}'(0,t)^2 + g_{n3}(0,t)^2)^{\alpha /2}, $$

$$\begin{aligned} \phi _n(x, t, 1) = (K_ng_{n1}(x_{n+2}, t)x_{n+1}, g_{n2}(x_{n+2}, t), g_{n3}(x_{n+2}, t)x_{n+3}), \end{aligned}$$

with the data

$$ \omega _n(x, 1) = A^{R^n}\prod _{i=1}^3\psi (L^{R^n}x_i)\sin (N^{R^n}x_{n+2})\sin (N^{R^n}x_{n+3})e_{n+1} + \cdots e_{n+3}, $$

where the subscripts on the right-hand side are taken modulo 3, and the coefficient of $e_{n+3}$ is chosen to make $\omega _n$ divergence-free, see (11).

### Lemma 13

For all $t \in [T_n, 1]$; the $e_{n+2}$ component of $\omega _n$ vanishes.

### Proof

Let $\omega$ be that component and *u* be the $e_{n+2}$ component of $U_n$. Then $\omega$ vanishes at time 1 and satisfies

$$ \partial _t\omega + U_n\cdot \nabla \omega = \omega _n\cdot \nabla u = \omega \partial _{n+2}u - g_n(t)\omega $$

because *u* depends only on $x_{n+2}$. This is a linear transport equation in terms of $\omega$, so the lemma follows from the uniqueness theorem. $\square$

For $i \not \equiv n + 2 \pmod 3$, the *i*-th component of the transport equation is

$$\begin{aligned} \partial _t(\omega _n)_i + U_n\cdot \nabla (\omega _n)_i&= \omega _n\cdot \nabla (U_n)_i - g_n(t)\omega _n\\&= (\omega _n)_i\partial _i(U_n)_i - g_n(t)\omega _{n}, \end{aligned}$$

because the *i*-th component of $U_n$, depends only on $x_i$ and $x_{n+2}$, and only the partial derivative in $x_i$ matters thanks to the lemma above. Then, by Lemmas 3 and 4,

13 $$\begin{aligned} \begin{aligned} \omega _n(x, t)&= G_n(t)K_n(t)^{-1}g_{n1}(x_{n+2}, t)^{-1}\omega _n(\phi _n(x, t, 1), 1)_{n+1}e_{n+1}\\&\quad + G_n(t)g_{n3}(x_{n+2}, t)^{-1}\omega _n(\phi _n(x, t, 1), 1)_{n+3}e_{n+3}\\&= G_n(t)K_n(t)^{-1}A^{R^n}\psi (L^{R^n}K_n(t)g_{n1}(x_{n+2}, t)x_{n+1})\psi (L^{R^n}g_{n2}(x_{n+2}, t))\\&\quad \times \sin (N^{R^n}g_{n2}(x_{n+2}, t))\psi (L^{R^n}g_{n3}(x_{n+2}, t)x_{n+3})\\&\quad \times \sin (N^{R^n}g_{n3}(x_{n+2}, t)x_{n+3})e_{n+1} + \cdots e_{n+3}, \end{aligned} \end{aligned}$$

where $$\phi _n(x, t, 1) = (K_ng_{n1}(x_{n+2}, t)x_{n+1}, g_{n2}(x_{n+2}, t), g_{n3}(x_{n+2}, t)x_{n+3})$$,

$$\begin{aligned} G_n(t)&= \exp \int _t^1 g_n(s)\text {d}s,&K_n(t)&= \int _t^1 -\partial _{n+2}(U_n)_{n+2}(0, 0, 0, s)\text {d}s, \end{aligned}$$

and the coefficient of $e_{n+3}$ makes $\omega _n(x, t)$ divergence free.

We will apply the estimates in Section 3 with $L_1 = K_n(t)L^{R^n}$, $L_2 = L_3 = L^{R^n}$ and $g_i = g_{ni}(\cdot , t)$ ($i = 1, 2, 3$) to get bounds on the velocity fields $\omega _n$ generates, which in turn dictates how the next vortex layer evolves according to the estimates in Section 2. Specifically, we will use Lemmas 1 and 2 to estimate $g_2$ for the next vortex layer and Lemma 3 to estimate $g_1$ and $g_3$ for the next vortex layer.

### The Induction

Note that $\omega _0$ is independent of *t*, so we have $G_0(t) = K_0(t) = g_{01} = g_{03} = g_{02}' = 1$ for all $t \in [0, 1]$. Let $T_n^* = 1 - 34R^nA^{-R^{n-1}}\ln (AN^s)$. Assume, as the induction hypothesis, that for all $0 \le k \le n - 1$, and $t \in [T_{k+1}^*, 1]$ we have $1 \le G_k(t) \le K_k(t) \le 2$, $g_{k1}, g_{k3} \in [1/2, 2]$, $\partial _xg_{k2} \in [1/2, 1]$, $|\partial _xg_{k1}|$, $|\partial _xg_{k3}|$ and $|\partial _x^2g_{k2}| < (N^2/L^R)^{R^{k-1}}$ and that $|\partial _x^2g_{k1}|$ and $|\partial _x^2g_{k3}| < N^{2R^{k-1}}$. Then for $t \in [T_{k+1}^*, 1]$ we can apply the velocity estimates in Section 3 to $\omega _k$, with $M = N^{R^k}$, $L_2 = L_3 = L^{R^k}$ and $L_1 = K_k(t)L^{R^k} \le 2L^{R^k}$.

We first show that the strength of the saddle point of the velocity field $U_n$ (defined in (12)) at the origin is comparable to the magnitude of $\omega _{n-1}$, i.e., $A^{R^{n-1}}$.

#### Lemma 14

If *N* is sufficiently large then for $t \in [T_n^*, 1]$,

$$ -\partial _{n+2}(U_n)_{n+2}(0, 0, 0, t) \in (1/34, 35/34)A^{R^{n-1}}. $$

#### Proof

We have

$$\begin{aligned} B_0&= \sum _{i=1}^3 L_i + L_3\ln M + \sup |\partial _xg_{k1}| + ML_3^{-1}(\sup |\partial _xg_{k3}| + \sup |\partial _x^2g_{k2}|)\\&< L^{R^k}(4 + \ln M) + (1 + 2(N/L)^{R^k})(N^2/L^R)^{R^{k-1}}\\&\lesssim L^{R^k}\ln M + (N^{1+2/R}/L^2)^{R^k}. \quad {(\ln M \ge \ln N \gg 1, \,\,N/L \gg 1)} \end{aligned}$$

We now check that $L > N^{(1+2/R)/3}$. Indeed, $L = N^{1+\alpha -s-2\sqrt{2\alpha /7}} = N^{(2+\alpha )/4+\frac{3}{2}\sqrt{2\alpha /7}} = N^{1/3+(2+3\alpha )/12+\frac{3}{2}\sqrt{2\alpha /7}}> N^{\frac{1}{3}+\sqrt{14\alpha }/6+\frac{3}{2}\sqrt{2\alpha /7}} = \root 3 \of {N}^{1+8\sqrt{2\alpha /7}} > \root 3 \of {N}^{1+7\sqrt{2\alpha /7}} = N^{(1+2/R)/3}$. Then $N^{1+2/R}/L^2 < L$, so the first term dominates and we have

Since $L_2 = L_3 = L^{R^k} \le M^{5/6}$, and $L_1 \le 2L^{R^k} \le 2M^{5/6}$, we can apply Lemma 5 and Corollary 1 to get, for $t \in [T_{k+1}^*, 1]$ and $\delta \in (0, 1)$,

$$ \Vert \omega _k(t) * \mathcal {K}\Vert _{C^1} \lesssim _\delta A^{R^k}(1 + B_0M^{\delta -1}). $$

Let $r = g_{k3}/\partial _xg_{k2}$. Then $r \in [1/2, 4]$, so

$$ \frac{G_k(t)(\partial _xg_{k2})g_{k3}}{K_k(t)g_{k1}(g_{k3}^2 + (\partial _xg_{k2})^2)} \in \frac{[1/4, 2]}{r + 1/r} \in [1/17, 1]. \quad {(r = g_{k3}/g_{k2}' \in [1/2, 4])} $$

By Lemma 8, for $i \equiv k \pmod 3$,

$$ -\partial _i(\omega _k * \mathcal {K})_i(0, 0, 0, t) \in [1/17, 1]A^{R^k} \pm O_\delta (A^{R^k}B_0M^{\delta -1}). $$

Since $$B_0 \lesssim L^{R^k}\ln M \lesssim _\delta M^{5/6+\delta }$$,

$$ -\partial _i(\omega _k * \mathcal {K})_i(0, 0, 0, t) \in [1/17, 1]A^{R^k} \pm O_\delta (A^{R^k}M^{2\delta -1/6}). $$

Let $\delta \le 0.08$. For $k = n - 1$, $M^{2\delta -1/6} \le N^{2\delta -1/6}$ has a negative exponent, so if *N* is sufficiently large then for $t \in [T_n^*, 1]$,

14 $$\begin{aligned} -\partial _{n+2}(\omega _{n-1} * \mathcal {K})_{n+2}(0, 0, 0, t) \in (3/68, 69/68)A^{R^{n-1}}. \end{aligned}$$

For $$0 \le k \le n - 2$$ and $$t \in [T_{k+1}^*, 1]$$,

$$ \Vert \omega _k(t) * \mathcal {K}\Vert _{C^1} \lesssim A^{R^k}(1 + M^{2\delta -1/6}) \lesssim A^{R^k} $$

so for $t \in [T_n^*, 1]$, if $A = N^{\sqrt{2\alpha /7}}$ is sufficiently large then

15 $$\begin{aligned} \sum _{k=0}^{n-2} \Vert \omega _k(t) * \mathcal {K}\Vert _{C^1} \lesssim \sum _{k=0}^{n-2} A^{R^k} {\left\{ \begin{array}{ll} \lesssim A^{R^{n-2}}, &  n \ge 2\\ = 0, &  n = 1 \end{array}\right. } < A^{R^{n-1}}/68. \end{aligned}$$

Since $\Omega = \sum _{k=0}^{n-1} \omega _k$, and $U_n$ agrees with $\Omega * \mathcal {K}$ on the $x_{n+2}$ axis by (12), the result follows from (14) and (15). $\square$

Next we want to find a subinterval $[T_n, 1] \subset [T_n^*, 1]$ in which the assumptions needed to apply the flow map estimates in Section 2 hold. For that purpose we need to estimate higher derivatives. Since $\nabla U_n$ has magnitude $A^{R^{n-1}}$ and frequency $N^{R^{n-1}}$, we expect its *k*-th derivative to be of order $(AN^k)^{R^{n-1}}$.

#### Lemma 15

There is a constant $C > 1$ such that if *N* is sufficiently large then for $t \in [T_n^*, 1]$ and $i = 1, 2, 3$, $\Vert \partial _i(U_n)_i(t)\Vert _{C^2} \le C(AN^2)^{R^{n-1}}$.

#### Proof

For $0 \le k \le n - 1$, there are two values of $i \in \{1, 2, 3\}$ such that $\partial _i\omega _k$ is of the same form as $\omega _k$, with an extra factor being $L_1g_{k1}(x_{k+2})$, $L_3g_{k3}(x_{k+2})$ or $Mg_{k3}(x_{k+2})$, so for these two values of *i* and $t \in [T_{k+1}^*, 1]$, by Corollary 2,

$$ \Vert \partial _i(\omega _k(t) * \mathcal {K})_i\Vert _{C^2} \lesssim _\delta A^{R^k}(M^2 + B_1M^\delta ). $$

As $\omega _k * \mathcal {K}$ is divergence free, the above then holds for all $i = 1, 2, 3$. Since, as showed in the proof of Lemma 14, $B_0 \lesssim L^{R^k}\ln M \lesssim _\delta M^{5/6+\delta } \le M$ for *M* sufficiently large,

$$\begin{aligned} B_1&= B_0(M + B_0) + \sup |\partial _x^2g_{k1}| + ML_3^{-1}\sup |\partial _x^2g_{k3}|\\&\lesssim MB_0 + (1 + (N/L)^{R^k})N^{2R^{k-1}} \lesssim ML^{R^k}\ln M + L^{2R^k} \lesssim ML^{R^k}\ln M \end{aligned}$$

where we have used the bound $N^{1+2/R}/L < L^2$ proven before. Since $B_1 \lesssim _\delta M^{11/6+\delta }$ and $M \gg 1$, for $t \in [T_{k+1}^*, 1]$ and $i = 1, 2, 3$,

$$ \Vert \partial _i(\omega _k(t) * \mathcal {K})_i\Vert _{C^2} \lesssim A^{R^k}M^2 = (AN^2)^{R^k}. $$

Summing from 0 to $n - 1$ and noting that $\partial _i(U_n)_i(x, t) = \partial _i(\Omega _n * \mathcal {K})_i(x_{n+2}e_{n+2}, t)$ gives the result. $\square$

Let $X = N^{-R^{n-1}}P^{-R^n}$, where $P = 6C\ln A$, with *C* given in Lemma 15. We check that this choice of *X* allows us to apply the lemmas in Section 2.

#### Lemma 16

If $A > e$ and $t \in [T_n^*, 1]$, then $X^2\Vert \partial _{n+2}(U_n)_{n+2}(\cdot ,t)\Vert _{C^2} < -\partial _{n+2}(U_n)_{n+2}(0, 0, 0, t)$.

#### Proof

Using $X^2 \le N^{-2R^{n-1}}P^{-2R^n}$ and Lemma 15 we get,

16 $$\begin{aligned} X^2\Vert \partial _{n+2}(U_n)_{n+2}(\cdot ,t)\Vert _{C^2} \le CA^{R^{n-1}}P^{-2R^n}. \end{aligned}$$

Since *R*, *C* and $\ln A > 1$, $P^{2R^n}> P^2 > 36C$, so the right-hand side is less than $A^{R^{n-1}}/36$. Comparing this bound with the one given by Lemma 14 shows the claim. $\square$

By Lemma 14, there is $T_n$ such that $1 - T_n \in (34/35, 34)R^nA^{-R^{n-1}}\ln (AN^s)$ (in particular $T_n \in [T_n^*, 1]$) and

17 $$\begin{aligned} \ln K_n(T_n) = \int _{T_n}^1 -\partial _{n+2}(U_n)_{n+2}(0, 0, 0, s)\text {d}s = R^n\ln (AN^s), \end{aligned}$$

which we will choose as the definition of $T_n$.

Recall that $\phi _n$ is the flow map of the velocity field $U_n$ defined in (12).

#### Lemma 17

If *N* is sufficiently large (so is $A = N^{\sqrt{2\alpha /7}}$) then for $x \in {{\,\textrm{supp}\,}}\omega _n(\cdot , 1)$ and $t \in [T_n, 1]$, $|\phi _n(x, 1, t)_{n+2}| \le X$.

#### Proof

Since $K_n(t){\le }K_n(T_n){=}(AN^s)^{R^n}$, we have $K_n(t)^{-1}X{\ge }(AN^{1/R+s}P)^{{-}R^n}$. By (16),

$$ \int _t^1 \frac{X^2\Vert \partial _{n+2}(U_n)_{n+2}(s)\Vert _{C^2}}{6}\text {d}s \le \frac{C(1 - T_n)}{6P^{2R^n}}A^{R^{n-1}}< cR^n\ln (AN^s). \quad {(c = 6C/P^2 < 1/\ln A)} $$

Then the exponential of the integral is less than $(A^cN^{cs})^{R^n}$, so

$$ K_n(t)^{-1}Xe^{\int _t^1 -\frac{X^2\Vert \partial _{n+2}(U_n)_{n+2}(s)\Vert _{C^2}}{6}\text {d}s} > (A^{1+c}N^{1/R+s+cs}P)^{-R^n}. $$

We now check that $L > AN^{1/R+s}$. Indeed, via the logarithm it is equivalent to $1 + \alpha - s - 2\sqrt{2\alpha /7} < \sqrt{2\alpha /7} + \sqrt{7\alpha /2} + s$. After substituting *s* and rearranging, this is equivalent to $\sqrt{2\alpha /7} > \alpha$, which is true because $\alpha < 2/7$. As $A \rightarrow \infty$, $c < 1/\ln A \rightarrow 0$, so the above inequality (which is really between the exponents) can be improved to $L > A^{1+c}N^{1/R+s+cs}$. Also thanks to the margin in the exponent, together with the fact that $P \lesssim \ln A$, the inequality can be further improved to $L > A^{1+c}N^{1/R+s+cs}P$. Then on supp $\omega _n(\cdot , 1)$ we have,

$$ |x_{n+2}| \le L^{-R^n} < K_n(t)^{-1}Xe^{\int _t^1 -\frac{X^2\Vert \partial _{n+2}(U_n)_{n+2}(s)\Vert _{C^2}}{6}\text {d}s}. $$

Then the claim follows from Lemma 2 (i). $\square$

Next we record a useful limit.

#### Lemma 18

If $r > 1$ then $ra^r$ and $ra^r\ln a \rightarrow 0$ as $a \rightarrow 0+$, uniformly in *r*.

#### Proof

We have $\partial _r(ra^r) = (1 + r\ln a)a^r < 0$ when $r > 1/2$ and $a < 1/e^2$. Then $ra^r < \sqrt{a}/2 \rightarrow 0$ as $a \rightarrow 0+$, uniformly in $r > 1/2$. Moreover, for $r>1$ we have $|\ln a| = \ln (1/a) = 2\ln (1/\sqrt{a})< 2/\sqrt{a} < 2/\sqrt{a^r}$, so $|ra^r\ln a| < 2ra^{r/2} = 4(r/2)a^{r/2} \rightarrow 0$ as $a \rightarrow 0+$, uniformly in $r > 1$. $\square$

Now we close the induction hypothesis for *n*, using the bounds from the flow map estimates in Section 2.

#### Lemma 19

(i) For $$t\in [T_{n+1}^*,1]$$, $$1\le K_n(t)<2$$.
(ii) For $t\in [T_n,1]$, $|g_{n2}'|\le 1$, $g_{n1}$ and $g_{n3}\in (1/2,2)$.
(iii) For $t\in [T_{n+1}^*,1]$, $|\partial _xg_{n1}|$, $|\partial _xg_{n3}|$ and $|\partial _x^2g_{n2}|<(N^2/L^R)^{R^{n-1}}$.
(iv) For $t\in [T_{n+1}^*,1]$, $|\partial _x^2g_{n1}|$ and $|\partial _x^2g_{n3}|<N^{2R^{n-1}}$.
(v) If $\nu \le 0.01$ then for $t\in [T_n,1]$, $1\le G_n(t)\le K_n(t)$.

#### Remark 3

Recall that

$$\begin{aligned} 1 - T_n&\in (34/35, 34)R^nA^{-R^{n-1}}\ln (AN^s),\\ 1 - T_{n+1}^*&= 34R^{n+1}A^{-R^n}\ln (AN^s), \end{aligned}$$

so

$$ \frac{1 - T_{n+1}^*}{1 - T_n}< 35RA^{R^{n-1}-R^n} < 35RA^{1-R} = 35R(A^{(1-R)/R})^R. $$

Since $R > \sqrt{2}$, as $A \rightarrow +\infty$, $A^{(1-R)/R} \rightarrow 0+$, uniformly in *R*. Then the right-hand side $\rightarrow 0$, also uniformly in *R* by Lemma 18. In particular it can be made smaller than 1. Then $T_{n+1}^* > T_n$, so (ii) and (v) also hold on $[T_{n+1}^*, 1]$, the time interval needed to recover the induction hypothesis.

#### Proof

(i) For $t \in [T_{n+1}^*, 1]$ we have

$$\begin{aligned} 0&\le \ln K_n(t) = \int _t^1 -\partial _{n+2}(U_n)_{n+2}(0, 0, 0, s)\text {d}s \lesssim (1 - T_{n+1}^*)A^{R^{n-1}}\\&\lesssim R^{n+1}A^{R^{n-1}-R^n}\ln (AN^s) \lesssim R^{n+1}(A^{(1-R)/R^2})^{R^{n+1}}\ln A. \end{aligned}$$

Since $R > \sqrt{2}$, as $A \rightarrow +\infty$, $A^{(1-R)/R^2} \rightarrow 0+$, uniformly in *R*. Then the right-hand side $\rightarrow 0$, uniformly in *R* and *n* by Lemma 18. In particular it can be made smaller than ln2.

(ii) By Lemma 2 (ii), $|g_{n2}'| \le 1$. By Lemma 8, for $0 \le k \le n - 1$ and $t \in [T_{k+1}^*, 1]$,

$$ |\nabla (\omega _k(t) * \mathcal {K})_{k+1}| \lesssim _\delta A^{R^k}L_1(M + B_0)M^{\delta -2} \lesssim A^{R^k}L_1M^{\delta -1}. $$

Since $L_1 \le 2L^{R^k}$ and $AL = N^{1+\alpha -s-\sqrt{2\alpha /7}} = N^{(2+\alpha )/4+2.5\sqrt{2\alpha /7}}$ with an exponent less than 1, taking $\delta$ sufficiently small we have that $|\nabla (\omega _k(t) * \mathcal {K})_{k+1}| \lesssim 1$. In particular, this holds for $\partial _n(\omega _{n-1}(t) * \mathcal {K})_n$ on the time interval $[T_n^*, 1]$. On the other hand,

$$ \sum _{k=0}^{n-2} \Vert \omega _k * \mathcal {K}\Vert _{C^1} \lesssim \sum _{k=0}^{n-2} A^{R^k} \lesssim A^{R^{n-2}}, $$

so, for $$t \in [T_n^*, 1]$$,

$$ |\partial _n(U_n(t))_n| = |\partial _n(\Omega _n(t) * \mathcal {K})_n| \lesssim A^{R^{n-2}}, $$

$$\begin{aligned} \int _t^1 |\partial _n(U_n)_n(s)|\text {d}s \lesssim R^nA^{R^{n-2}-R^{n-1}}\ln (AN^s)\\ \lesssim R^n(A^{\frac{1-R}{R^2}})^{R^n}\ln A \rightarrow 0 \end{aligned}$$

as $A \rightarrow \infty$, uniformly in *R* and *n* by Lemma 18. Also, by (16),

18 $$\begin{aligned} \begin{aligned} \int _t^1 \frac{X^2\Vert \partial _{n+2}(U_n)_{n+2}(s)\Vert _{C^2}}{2}\text {d}s&\lesssim \frac{1 - T_n}{P^{2R^n}}A^{R^{n-1}} \lesssim \frac{R^n\ln (AN^s)}{P^{2R^n}}\\&\lesssim R^n(\ln A)^{1-2R^n} \le R^n(\ln A)^{-R^n} \rightarrow 0 \end{aligned} \end{aligned}$$

as $A \rightarrow \infty$, uniformly in *R* and *n* by Lemma 18. Then by Lemma 3, when *A* is sufficiently large, $|\ln g_{n3}|$ and $|\ln g_{n1}| < \ln 2$ for $t \in [T_n, 1]$,

(iii) By Lemma 1 (iv), for $t \in [T_{n+1}^*, 1]$ and $|x_2| \le X$,

$$ |\phi _n(x, t, 1)_{n+2}| \ge K_n(t)^{-1}|x_{n+2}|\exp \int _t^1 -\frac{X^2\Vert \partial _{n+2}(U_n)_{n+2}(s)\Vert _{C^2}}{6}\text {d}s \gtrsim |x_{n+2}|, $$

so, for $x \in {{\,\textrm{supp}\,}}\omega _n(\cdot , 1)$, $|\phi _n(x, 1, t)_{n+2}| \lesssim |x_{n+2}| \lesssim L^{R^{-n}}$. By Lemma 15, for $i = 1, 2, 3$,

$$ \Vert \partial _i(U_n)_i(t)\Vert _{C^2} \lesssim (AN^2)^{R^{n-1}} $$

so

$$\begin{aligned} |\phi _n(x, 1, t)_{n+2}|\int _t^1 \Vert \partial _i(U_n)_i(s)\Vert _{C^2}\text {d}s&\lesssim R^{n+1}A^{R^{n-1}-R^n}\ln (AN^s)(N^2/L^R)^{R^{n-1}}\\&< (N^2/L^R)^{R^{n-1}}/4 \end{aligned}$$

when $A = N^{\sqrt{2\alpha /7}}$ is sufficiently large. Then by Lemmas 2 and 3, $|\partial _x^2g_{n2}|$, $|\partial _x\ln g_{n3}|$ and $|\partial _x\ln g_{n1}| < (N^2/L^R)^{R^{n-1}}/2$. Since $|g_{n1}|$, $|g_{n3}| < 2$, we have $|\partial _xg_{n3}|$, $|\partial _xg_{n1}| < (N^2/L^R)^{R^{n-1}}$ for $t \in [T_{n+1}^*, 1]$.

(iv) Similarly, for $t \in [T_{n+1}^*, 1]$ and $i = 1, 2, 3$, when $A = N^{\sqrt{2\alpha /7}}$ is sufficiently large,

$$ \int _t^1 \Vert \partial _i(U_n)_i(s)\Vert _{C^2}\text {d}s \lesssim R^{n+1}A^{R^{n-1}-R^n}\ln (AN^s)N^{2R^{n-1}} < N^{2R^{n-1}}/8. $$

Since $K_n(t) \le 2$, $1 + \ln K_n(t) \le 2$, so by Lemma 3, $|\partial _x^2\ln g_{n3}|$ and $|\partial _x^2\ln g_{n1}|<N^{2R^{n-1}}/4$. Then for $i = 1, 3$,

$$ |\partial _x^2g_{ni}/g_{ni}| \le |\partial _x^2\ln g_{ni}| + (\partial _xg_{ni}/g_{ni})^2 = |(\ln g_{ni})''| + ((\ln g_{ni})')^2 < N^{2R^{n-1}}/2. $$

Since $|g_{n1}|$ and $|g_{n3}| < 2$, we have $|\partial _x^2g_{n3}|$ and $|\partial _x^2g_{n1}| < N^{2R^{n-1}}$ for $t \in [T_{n+1}^*, 1]$.

(v) For $t \in [T_n, 1]$ we have $g_n(t) = \nu N^{\alpha R^n}((\partial _xg_{n2}(0))^2 + g_{n3}(0)^2)^{\alpha /2} \ge 0$. On the other hand, since $\nu \le 0.01$, $|\partial _xg_{n2}(0)|\le 1$, $|g_{n3}(0)|<2$ and $\alpha <1/9$, we have $g_n(t)< 0.01\root 18 \of {5}N^{\alpha R^n}< 0.02N^{\alpha R^n} = 0.02A^{R^{n-1}} < -\partial _{n+2}(U_n)_{n+2}(0, 0, 0, t)$, so integrating gives the result. $\square$

### Estimating $\omega _n$

By Lemma 19 (ii), $|\ln g_{n1}|$ and $|\ln g_{n3}| < \ln 2$ for $t \in [T_n, 1]$. Since $K_n(t) \le K_n(T_n) = (AN^s)^{R^n}$, by Lemma 1 (iv) and (18), for $|x_2| \le X$,

$$\begin{aligned} |\phi _n(x, t, 1)_{n+2}|&\ge K_n(t)^{-1}|x_{n+2}|\exp \int _t^1 -\frac{X^2\Vert \partial _{n+2}(U_n)_{n+2}(s)\Vert _{C^2}}{6}\text {d}s,\\&\gtrsim K_n(t)^{-1}|x_{n+2}| \end{aligned}$$

so, for $x \in {{\,\textrm{supp}\,}}\omega _n(\cdot , 1)$, $|\phi _n(x, 1, t)_{n+2}| \lesssim K_n(t)L^{-R^n}$. Then by Lemma 15, for $i = 1, 2, 3$,

$$ |\phi _n(x, 1, t)_{n+2}|\Vert \partial _i(U_n)_i(t)\Vert _{C^2} \lesssim K_n(t)(AN^2/L^R)^{R^{n-1}}. $$

Since $$1 - T_n \lesssim R^nA^{-R^{n-1}}\ln (AN^s) \lesssim R^nA^{-R^{n-1}}\ln N$$,

$$ |\phi _n(x, 1, t)_{n+2}|\int _t^1 \Vert \partial _i(U_n)_i(s)\Vert _{C^2}\text {d}s \lesssim K_n(t)R^n(N^2/L^R)^{R^{n-1}}\ln N. $$

Since $X^2\Vert \partial _{n+2}(U_n)_{n+2}(t)\Vert _{C^2} < -\partial _{n+2}(U_n)_{n+2}(0, 0, 0, t)$ and $|\phi _n(x, 1, t)_{n+2}| < X$, Lemma 2 and 3 apply to give

$$ |\partial _x^2g_{n2}|, |\partial _x\ln g_{n1}|, |\partial _x\ln g_{n3}| \lesssim K_n(t)R^n(N^2/L^R)^{R^{n-1}}\ln N. $$

Since $|g_{n1}|, |g_{n3}| \le 2$, $|\partial _xg_{n1}|$ and $|\partial _xg_{n3}|$ have the same bound. Also, for $i = 1, 2, 3$,

$$ \int _t^1 \Vert \partial _i(U_n)_i(s)\Vert _{C^2}\text {d}s \lesssim R^nN^{2R^{n-1}}\ln N, $$

so, using Lemma 3 and $K_n(t) \le (AN^s)^{R^n} = N^{(\sqrt{2\alpha /7}+s)R^n}< N^{(3\alpha +2)R^n/4} < N^{R^n}$,

$$ |\partial _x^2\ln g_{n1}|, |\partial _x^2\ln g_{n3}| \lesssim (1 + \ln K_n(t))R^nN^{2R^{n-1}}\ln N \lesssim R^nN^{2R^{n-1}}\ln ^2N. $$

When $t \in [T_n, 1]$, for $\omega _n$ we have $M = N^{R^n}$, $L_2 = L_3 = L^{R^n}$, $L_1 = L^{R^n}K_n(t)$. Then

$$\begin{aligned} B_0&= \sum _{i=1}^3 L_i + L_3\ln M + \sup |\partial _xg_{n1}| + ML_3^{-1}(\sup |\partial _xg_{n3}| + \sup |\partial _x^2g_{n2}|)\\&\lesssim K_n(t)L^{R^n}\ln M + K_n(t)(N^{1+2/R}/L^2)^{R^n}\ln M\\&\lesssim K_n(t)L^{R^n}\ln M, \end{aligned}$$

because the first term dominates as before.

Since $L = N^{1+\alpha -s-2\sqrt{2\alpha /7}} = N^{1-s+\alpha -\sqrt{2\alpha /7}}/A$, we have

$$ K_n(t)L^{R^n} \le (ALN^s)^{R^n} = N^{(1+\alpha -\sqrt{2\alpha /7})R^n} = M^{1+\alpha -\sqrt{2\alpha /7}}. $$

Moreover $\alpha < 2/7$, so Lemmas 5 and 6 apply and $B_0 \lesssim M$. Then

$$\begin{aligned} B_1&= B_0(M + B_0) + \sup |\partial _x^2g_{n1}| + ML_3^{-1}\sup |\partial _x^2g_{n3}|\\&\lesssim MB_0 + (N/L)^{R^n}N^{2R^{n-1}}\ln ^2M\\&\lesssim K_n(t)ML^{R^n}\ln M + L^{2R^n}\ln ^2M \le 2K_n(t)ML^{R^n}\ln M, \le 2M^2\ln M \end{aligned}$$

because $K_n(t) \ge 1$ and $L^{R^n}\ln ^2M< M^{5/6}\ln ^2M < M$ when $M \gg 1$.

### Estimating the Force

In our construction, the vorticity is an infinite sum whose terms are turned on at different time scales:

$$ \omega (x, t) = \sum _{n=0}^\infty \rho _n(t)\omega _n(x, t). $$

Here $\rho _n$ is supported on $[T_n, 1]$, has values in [0, 1], and is a smooth approximation of $1_{[T_n,1]}$ in $L^1$. By (13), each $\omega _n$ is smooth in (*x*, *t*). Since

$$ 1 - T_n < 34R^nA^{-R^{n-1}}\ln (AN^s) \rightarrow 0 \quad {(n \rightarrow \infty )} $$

on any compact subset of [0, 1), only finitely many $\omega _n$ is non-zero, so the whole sum is smooth in $(x, t) \in \mathbb {R}^3 \times [0, 1)$, with

$$ \partial _t\omega (x, t) = \sum _{n=0}^\infty (\rho _n'(t)\omega _n(x, t) + \rho _n(t)\partial _t\omega _n(x, t)), $$

so

$$ \partial _t\omega + (\omega * \mathcal {K})\cdot \nabla \omega - \omega \cdot \nabla (\omega * \mathcal {K}) - \nu |\nabla |^\alpha \omega = \sum _{n=0}^\infty F_{n}, $$

where the sum is smooth in $(x, t) \in \mathbb {R}^3 \times [0, 1)$, and the summand

$$\begin{aligned} F_n&= \rho _n(t)F_n^\circ (x, t) + \rho _n'(t)\omega _n(x, t),\\ F_n^\circ&= (\omega _n * \mathcal {K})\cdot \nabla \omega _n - \omega _n\cdot \nabla (\omega _n * \mathcal {K})\\&\quad + (\omega _n * \mathcal {K})\cdot \nabla \Omega _n - \Omega _n\cdot \nabla (\omega _n * \mathcal {K})\\&\quad + (\Omega _n * \mathcal {K} - U_n)\cdot \nabla \omega _n - \omega _n\cdot \nabla (\Omega _n * \mathcal {K} - U_n)\\&\quad + g_n(t)\omega _n - \nu |\nabla |^\alpha \omega _n\\&\quad + \partial _t\omega _n + U_n\cdot \nabla \omega _n - \omega _n\cdot \nabla U_n - g_n(t)\omega _n \quad {(\textrm{This}\,\, \textrm{line} \,\, \textrm{equals}\,\, \textrm{0}\,\, \textrm{by}\,\, \mathrm{construction.})} \end{aligned}$$

will be controlled in the $C^r$ norm, where $r \in (0, s)$, which is possible because $s > 0$:

$$ \Vert F_n\Vert _{L_t^1C_x^r} \le \Vert \rho _n(t)F_n^\circ \Vert _{L_t^1C_x^r} + \Vert \rho _n'(t)\omega _n\Vert _{L_t^1C_x^r}. $$

Since $\rho _n$ is supported on $[T_n, 1]$ and has values in [0, 1],

$$ \Vert \rho _n(t)F_n^\circ \Vert _{L_t^1C_x^r} \le \int _{T_n}^1 \Vert F_n^\circ (t)\Vert _{C^r}dt. $$

Since $\omega _n$ is continuous in $t \in [T_n, 1]$,

$$ \Vert \rho _n'(t)\omega _n\Vert _{L_t^1C_x^r} \rightarrow \Vert \omega _n(\cdot , T_n)\Vert _{C^{r}}, $$

as $\rho _n$ is taken to be arbitrarily close to $1_{[T_n,1]}$ in $L^1$. Thus to show that $\sum _{n=0}^\infty F_n$ is finite in $L_t^1C_x^r$ norm, it suffices to show the finiteness of the sums $\sum _{n=0}^\infty \Vert \omega _n(\cdot , T_n)\Vert _{C^r}$ and $\sum _{n=0}^\infty \int _{T_n}^1 \Vert F_n^\circ (t)\Vert _{C^r}dt$. The former is shown in Section 4.3.1, while the latter, itself containing multiple terms, are shown in Sections 4.3.2 to 4.3.5.

#### Switching on $\omega _n$ at Time $T_n$

Let $\nu = 0.005(s - r)$. For $t \in [T_n, 1]$ we have $g_n(t) = \nu N^{\alpha R^n}(\partial _xg_{n2}(0)^2 + g_{n3}(0)^2)^{\alpha /2}$, where $|\partial _xg_{n2}(0)|\le 1$, $|g_{n3}(0)|<2$ and $\alpha <1/9$, so $g_n(t)< \nu \root 18 \of {5}N^{\alpha R^n} < 0.01(s-r)N^{\alpha R^n} = 0.01(s-r)A^{R^{n-1}}$, so

$$\begin{aligned} G_n(t)&= \exp \int _t^1 g_n(s)\text {d}s \le e^{0.01(s-r)A^{R^{n-1}}T_n}< e^{0.4(s-r)R^n\ln (AN^s)}\\&= N^{0.4(s-r)R^n(s+\sqrt{2\alpha /7})} < N^{0.4(s-r)R^n}, \end{aligned}$$

because $s + \sqrt{2\alpha /7}< (3\alpha + 2)/4 + \sqrt{2\alpha /7} < 1$ when $\alpha< 1/9 < 2/7$. Since $\sup |\omega _n(\cdot , t)| \lesssim G_n(t)K_n(t)^{-1}A^{R^n}$ and

$$ \Vert \omega _n(\cdot , t)\Vert _{C^1} \lesssim G_n(t)K_n(t)^{-1}A^{R^n}(M + B_0) \lesssim G_n(t)K_n(t)^{-1}A^{R^n}M $$

for $r \in (0, s)$, $\Vert \omega _n(\cdot , t)\Vert _{C^r} \lesssim G_n(t)K_n(t)^{-1}A^{R^n}M^r$. In particular, since

$$ K_n(T_n) = \exp \int _{T_n}^1 -\partial _{n+2}U_{n+2}(0, 0, 0, s)\text {d}s = (AN^s)^{R^n} = A^{R^n}M^s, $$

$$\Vert \omega _n(\cdot , T_n)\Vert _{C^r} \lesssim G_n(T_n)M^{r-s} < N^{(r-s)R^n/2}$$, so using $$r<s$$,

$$ \sum _{n=0}^\infty \Vert \omega _n(\cdot , T_n)\Vert _{C^r} < \infty . $$

#### The Quadratic Self-Interaction

By Lemma 7, for $r \in (0, s)$,

$$\begin{aligned} \Vert (\omega _n * \mathcal {K}) \cdot \nabla \omega _n\Vert _{C^r}&+ \Vert \omega _n \cdot \nabla (\omega _n * \mathcal {K})\Vert _{C^r}\\&\lesssim _\delta G_n(t)^2K_n(t)^{-2}A^{2R^n}B_0^{1-r}B_1^rM^{-2+2\delta }(M + B_0)\\&\lesssim _\delta G_n(t)^2K_n(t)^{-2}A^{2R^n}B_0^{1-r}B_1^rM^{-1+2\delta }\\&\lesssim _\delta A^{2R^n}L^{R^n}M^{r+0.4(s-r)-1+2\delta }\ln M, \end{aligned}$$

where in the last line we used $B_j \lesssim K_n(t)M^jL^{R^n}\ln M$ ($j = 0, 1$) and Lemma 19 (v), so

$$\begin{aligned} \int _{T_n}^1 (\Vert (\omega _n * \mathcal {K}) \cdot \nabla \omega _n\Vert _{C^r}&+ \Vert \omega _n \cdot \nabla (\omega _n * \mathcal {K})\Vert _{C^r})dt\\&\lesssim _\delta (1 - T_n)A^{2R^n}L^{R^n}M^{r+0.4(s-r)-1+2\delta }\ln M\\&\lesssim _\delta A^{2R^n-R^{n-1}}L^{R^n}M^{r+0.4(s-r)-1+2\delta }\ln ^2M\\&\lesssim _\delta A^{2R^n-R^{n-1}}L^{R^n}M^{r+0.4(s-r)-1+3\delta }\\&= (N^{2\sqrt{2\alpha /7}-\alpha +r+0.4(s-r)-1+3\delta }L)^{R^n} = N^{(0.6(r-s)+3\delta )R^n}, \end{aligned}$$

because $L = N^{1+\alpha -s-2\sqrt{2\alpha /7}}$. Since $r < s$, we can let $\delta = (s - r)/6 > 0$ so that the integral is $O(a^{R^n})$ for some $a \in (0, 1)$, so

$$ \sum _{n=0}^\infty \int _{T_n}^1 (\Vert (\omega _n * \mathcal {K}) \cdot \nabla \omega _n\Vert _{C^r} + \Vert \omega _n \cdot \nabla (\omega _n * \mathcal {K})\Vert _{C^r})dt < \infty . $$

#### The Interaction Between Inner Velocity and Outer Vorticity

For $t \in [T_n, 1]$ and $k = 0, \dots , n - 1$, $\Vert \omega _k\Vert _{C^{j}} \lesssim (AN^j)^{R^k}$ ($j = 0, 1, 2$). Summing over *k* we get $\Vert \Omega _n\Vert _{C^{j}} \lesssim (AN^j)^{R^{n-1}}$ ($j = 0, 1, 2$). Then by Lemma 10, for $j = 0, 1$,

$$\begin{aligned} \Vert (\omega _n * \mathcal {K}) \cdot \nabla \Omega _n\Vert _{C^j}&\lesssim _\delta G_n(t)K_n(t)^{-1}A^{R^n}(M + B_0)\sum _{j_1+j_2=j}M^{(j_1-2)(1-\delta )}\Vert \Omega _n\Vert _{C^{j_2+1}}\\&\lesssim _\delta A^{R^n}M\sum _{j_1+j_2=j}M^{(j_1-2)(1-\delta )}(AN^{j_2+1})^{R^{n-1}}, \end{aligned}$$

where we have used $B_0 \lesssim M$ and $K_n(t) \ge 1$. Since $N^{R^{n-1}} = M^{1/R} \le M^{1-\delta }$, the sum is dominated by the summand with $j_1 = j$ and $j_2 = 0$, so

$$ \Vert (\omega _n * \mathcal {K}) \cdot \nabla \Omega _n\Vert _{C^j} \lesssim _\delta M^{1+(j-2)(1-\delta )}(A^{1+R}N)^{R^{n-1}}. $$

By interpolation, for $r \in (0, s)$,

$$ \Vert (\omega _n * \mathcal {K}) \cdot \nabla \Omega _n\Vert _{C^r} \lesssim _\delta M^{1+(r-2)(1-\delta )}(A^{1+R}N)^{R^{n-1}}. $$

Since for $t \in [T_n, 1]$, $g_{n1}$ and $g_{n3} > 1/2$, for $x \in {{\,\textrm{supp}\,}}\omega _n(\cdot , t)$ we have $|x_{n+1}|$ and $|x_{n+3}| < 2L^{-R^n}$. Also $\phi _n(x, t, 1) \in {{\,\textrm{supp}\,}}\omega _n(\cdot , 1)$, so $|x_2| = |\phi _n(\phi _n(x, t, 1), 1, t)_{n+2}| \lesssim K_n(t)L^{-R^n}$, which dominates the previous bound and is therefore a bound for $D = \max \{|x|: x \in {{\,\textrm{supp}\,}}\omega _n(\cdot , t)\}$. Then, by Lemma 10, for $r \in [0, 1)$,

$$\begin{aligned} \Vert \Omega _n \cdot \nabla (\omega _n * \mathcal {K})\Vert _{C^r}&\lesssim _{r,\delta } G_n(t)K_n(t)^{-1}A^{R^n}M^{1+(r-1)(1-\delta )}D\Vert \Omega _n\Vert _{C^1}\\&\lesssim _{r,\delta } G_n(t)A^{R^n}M^{1+(r-1)(1-\delta )}(1/L^{R^n})(AN)^{R^{n-1}}\ln M\\&\lesssim _{r,\delta } M^{r+0.4(s-r)+2\delta }L^{-R^n}(A^{1+R}N)^{R^{n-1}} \end{aligned}$$

dominating the bound above because $L \le N$. Then

$$\begin{aligned} \int _{T_n}^1 (\Vert (\omega _n * \mathcal {K}) \cdot \nabla \Omega _n\Vert _{C^r}&+ \Vert \Omega _n \cdot \nabla (\omega _n * \mathcal {K})\Vert _{C^r})dt\\&\lesssim _\delta (1 - T_n)M^{r+0.4(s-r)+2\delta }L^{-R^n}(A^{1+R}N)^{R^{n-1}}\\&\lesssim _\delta M^{r+0.4(s-r)+2\delta }(A/L)^{R^n}N^{R^{n-1}}\ln M\\&\lesssim _\delta M^{r+0.4(s-r)+3\delta }(A/L)^{R^n}N^{R^{n-1}} < M^{0.6(r-s)+3\delta } \end{aligned}$$

because $L > AN^{1/R+s}$. Since $r < s$, we can let $\delta = (s - r)/6 > 0$ so that the integral is $O(a^{R^n})$ for some $a \in (0, 1)$, so

$$ \sum _{n=0}^\infty \int _{T_n}^1 (\Vert (\omega _n * \mathcal {K}) \cdot \nabla \Omega _n\Vert _{C^r} + \Vert \Omega _n \cdot \nabla (\omega _n * \mathcal {K})\Vert _{C^r})dt < \infty . $$

#### The Interaction Between Outer Velocity and Inner Vorticity

By Lemma 11,

$$\begin{aligned} \Vert (\Omega _n * \mathcal {K} - U_n) \cdot \nabla \omega _n\Vert _{C^r}&+ \Vert \omega _n \cdot \nabla (\Omega _n * \mathcal {K} - U_n)\Vert _{C^r}\\&\lesssim _\delta G_n(t)K_n(t)^{-1}A^{R^n}(M^{r+1} + B_0^{1-r}B_1^r)\\&\times (1/L_1 + 1/L_3)^2D^{1-\delta }\Vert \Omega \Vert _{C^2}\\&\lesssim A^{R^n}M^{r+1}L^{-(3-\delta )R^n}(AN^2)^{R^{n-1}}. \end{aligned}$$

Since $$1 - T_n \lesssim A^{-R^{n-1}}\ln M$$,

$$\begin{aligned} \int _{T_n}^1 (\Vert (\Omega _n * \mathcal {K} - U_n) \cdot \nabla \omega _n\Vert _{C^r}&+ \Vert \omega _n \cdot \nabla (\Omega _n * \mathcal {K} - U_n)\Vert _{C^r})dt\\&\lesssim A^{R^n}M^{r+1}L^{-(3-\delta )R^n}N^{2R^{n-1}}\ln M\\&= (N^{8\sqrt{2\alpha /7}+r+1}L^{-(3-\delta )})^{R^n}\ln M. \end{aligned}$$

When $\delta \rightarrow 0$, the base tends to

$$\begin{aligned} N^{8\sqrt{2\alpha /7}+r+1}L^{-3}&= N^{8\sqrt{2\alpha /7}+r+1-3(1+\alpha -s-2\sqrt{2\alpha /7})}\\&= N^{2\sqrt{14\alpha }-2-3\alpha +r+3s}. \end{aligned}$$

Since $r \in (0, s)$, $r + 3s < 4s = 3\alpha + 2 - 2\sqrt{14\alpha }$, so the exponent is negative, so as before

$$ \sum _{n=0}^\infty \int _{T_n}^1 (\Vert (\Omega _n * \mathcal {K} - U_n) \cdot \nabla \omega _n\Vert _{C^r} + \Vert \omega _n \cdot \nabla (\Omega _n * \mathcal {K} - U_n)\Vert _{C^r})dt < \infty . $$

#### The Dissipation

For $x \in {{\,\textrm{supp}\,}}\omega _n(\cdot , t)$, we have $\phi _n(x, t, 1) \in {{\,\textrm{supp}\,}}\omega _n(\cdot , 1)$, so

$$ |x_{n+2}| = |\phi _n(\phi _n(x, t, 1), 1, t)_{n+2}| \lesssim K_n(t)L^{-R^n}. $$

Also $|\partial _x^2g_{n2}|$ and $|\partial _xg_{n3}| \lesssim K_n(t)(N^2/L^R)^{R^{n-1}}\ln M$. Also $\alpha + r< \alpha + s< \alpha + (3\alpha + 2)/4 < 1$ when $\alpha < 1/9$. Then by Lemma 19 (ii), Corollary 4 applies. If $\omega$ is the main component of $\omega _n$ normalized to have amplitude 1 then

$$\begin{aligned} \Vert |\nabla |^{\alpha }\omega&- M^\alpha (\partial _xg_{n2}(0)^2 + g_{n3}(0)^2)^{\alpha /2}\omega \Vert _{C^r}\\&\lesssim _{r,\alpha ,\delta } BM^{\alpha +(r-1)(1-\delta )} + M^{\alpha +r}|x_2|\sup (|\partial _xg_{n2}| + |\partial _xg_{n3}|)\\&\lesssim _{r,\alpha ,\delta } BM^{\alpha +(r-1)(1-\delta )} + M^{\alpha +r}K_n(t)^2(N/L^R)^{2R^{n-1}}\ln M \end{aligned}$$

Now we specialize to $a = 1/g_{n1}(\cdot , t)$, so $\sup |a'| \lesssim \sup |\partial _xg_1|$, and the respective bound has *B* replaced by $B_0 = B$ without the term $\sup |a'|$. Since $B_0 \lesssim K_n(t)L^{R^n}\ln M$,

$$\begin{aligned} \Vert |\nabla |^\alpha \omega&- M^\alpha (\partial _xg_2(0)^2 + g_3(0)^2)^{\alpha /2}\omega \Vert _{C^r}\\&\lesssim _{r,\alpha ,\delta } K_n(t)L^{R^n}(\ln M)M^{\alpha +(r-1)(1-\delta )}\\&+ M^{\alpha +r}K_n(t)^2(N/L^R)^{2R^{n-1}}\ln M. \end{aligned}$$

As before the corresponding bound for the other component is better, so for $t \in [T_n, 1]$, taking into account the amplitude of $\omega _n$,

$$\begin{aligned}&\Vert \nu |\nabla |^\alpha \omega _n - g_n(t)\omega _n\Vert _{C^r}\\&\quad \lesssim _{r,\alpha ,\delta } G_n(t)(AL)^{R^n}(\ln M)M^{\alpha +(r-1)(1-\delta )}\\&\quad \quad + G_n(t)A^{2R^n}M^{\alpha +r+s}(N/L^R)^{2R^{n-1}}\ln M \quad \quad {(K_n(t) \le (AN^s)^{R^n})}\\&\quad< M^{\sqrt{2\alpha /7}+1+\alpha -s-2\sqrt{2\alpha /7}+\alpha +(s-1)(1-\delta )}\ln M\\&\quad \quad {(G_n(t) < M^{0.4(s-r)} \le M^{(s-r)(1-\delta )}\,\,\, \textrm{when} \,\,\delta \le 0.6)}\\&\quad \quad + M^{2\sqrt{2\alpha /7}+\alpha +2s+2\sqrt{7\alpha /2}-2(1+\alpha -s-2\sqrt{2\alpha /7})}\ln M\\&\quad \le 2M^{2\alpha -\sqrt{2\alpha /7}+(1-s)\delta }\ln M. \end{aligned}$$

Since $$1 - T_n \lesssim A^{-R^{n-1}}\ln M = M^{-\alpha }\ln M$$,

$$ \int _{T_n}^1 \Vert \nu |\nabla |^\alpha \omega _n - g_n(t)\omega _n\Vert _{C^r}dt \lesssim _{r,\alpha ,\delta } M^{\alpha -\sqrt{2\alpha /7}+(1-s)\delta }\ln M. $$

Since $\alpha < 2/7$, when $\delta$ is sufficiently small, the exponent is negative, so

$$ \sum _{n=0}^\infty \int _{T_n}^1 \Vert \nu |\nabla |^\alpha \omega _n - g_n(t)\omega _n\Vert _{C^r}dt < \infty . $$

Combining the bounds above, we get

$$ \sum _{n=0}^\infty \Vert F_n\Vert _{L_1^tC_x^r} \le \sum _{n=0}^\infty \int _{T_n}^1 \Vert F_n^\circ (t)\Vert _{C^r}dt + \sum _{n=0}^\infty \Vert w_n(T_n)\Vert _{C^r} < \infty . $$

### Proof of Theorem 1

In the above, for $0 \le \alpha < \frac{1}{9}(22 - 8\sqrt{7})$ we have constructed a divergence free $\omega \in C^\infty (\mathbb {R}^3 \times [0, 1))$ supported in $B(1) \times [0, 1]$ such that

$$ \partial _t\omega + u \cdot \nabla \omega + \nu |\nabla |^\alpha \omega = \omega \cdot \nabla u + F $$

where $\nu >0$, $F \in C^\infty (\mathbb {R}^3 \times [0, 1)) \cap L_t^1([0,1])C_x^r$ with $r>0$, and

$$ u(x, t) = \frac{1}{4\pi }\int _{\mathbb {R}^3} \frac{\omega (y, t) \times (x - y)}{|x - y|^3}\text {d}y $$

satisfies $\nabla \cdot u = 0$ and $\nabla \times u = \omega$. Now we do a Hodge decomposition

$$ \partial _tu + u \cdot \nabla u + \nu |\nabla |^\alpha u = -\nabla p + f $$

where $$\nabla \cdot f = 0$$. Then

$$ \partial _t\omega + u \cdot \nabla \omega + \nu |\nabla |^\alpha \omega = \omega \cdot \nabla u + \nabla \times f $$

so $\nabla \times f = F$. Since $\nabla \cdot f = 0$, *f* can be recovered from *F* using the Biot–Savart law:

$$ f(x, t) = \frac{1}{4\pi }\int _{\mathbb {R}^3} \frac{F(y, t) \times (x - y)}{|x - y|^3}\text {d}y. $$

Since *F* is compactly supported in $\mathbb {R}^3 \times [0, 1]$ and smooth in *x* for $t < 1$, *f* is square integrable and smooth in *x* for $t < 1$. Since $F \in L_t^1([0,1])C_x^r$, by Schauder estimates $f \in L_t^1([0,1])C_x^{1,r}$.

For $t \in [T_n, 1]$ we have $\sup |\omega _n(\cdot , t)| \lesssim G_n(t)K_n(t)^{-1}A^{R^n} \le A^{R^n}$, and since the flow is incompressible, $|{{\,\textrm{supp}\,}}\omega _n(\cdot , t)| = |{{\,\textrm{supp}\,}}\omega _n(\cdot , 1)| \lesssim L^{-3R^n}$, so

$$ \Vert \omega _n(\cdot , t)\Vert _{L^{6/5}} \lesssim (AL^{-5/2})^{R^n},\ \Vert \omega _n(\cdot , t)\Vert _{L^4} \lesssim (AL^{-3/4})^{R^n}. $$

Since $$L > A^{3/2}$$,

$$ \sum _n\Vert \omega _n(\cdot , t)\Vert _{L^{6/5}} \lesssim \sum _nA^{-11R^n/4}< \infty ,\ \sum _n\Vert \omega _n(\cdot , t)\Vert _{L^4} \lesssim \sum _nA^{-R^n/8} < \infty $$

so $\omega = \sum _n\omega _n \in L_t^\infty (L_x^{6/5} \cap L_x^4)$, so $\nabla u = \omega * \nabla \mathcal {K}$ is also in this space. Then $u = \omega * \mathcal {K} \in L_t^\infty L_x^2$ by Sobolev embedding. Since $\nabla u$ is also in this space by interpolation between $L^{6/5}$ and $L^4$, $u \in L_t^\infty L_x^6$ again by Sobolev embedding. Then $u \in L_t^\infty L_x^4$ by interpolation. Since we have shown that $\nabla u$ is also in this space, $u \in L_{t,x}^\infty$ again by Sobolev embedding.

For $t \in [2T_{n+1} - 1, T_{n+1}]$ we have $\sup |\omega _n(\cdot , t)| \gtrsim G_n(t)K_n(t)^{-1}A^{R^n} \ge K_n(t)^{-1}A^{R^n}$. Since

$$\begin{aligned} 0&\le \ln K_n(t) = \int _t^1 -\partial _{n+2}(U_n)_{n+2}(0, 0, 0, s)\text {d}s \lesssim 2(1 - T_{n+1}^*)A^{R^{n-1}}\\&\lesssim R^{n+1}A^{R^{n-1}-R^n}\ln (AN^s) \lesssim R^{n+1}(A^{(1-R)/R^2})^{R^{n+1}}\ln A \rightarrow 0 \end{aligned}$$

as $A \rightarrow +\infty$, uniformly in *R* by Lemma 18, $\sup |\omega _n(\cdot , t)| \gtrsim A^{R^n}$. On the other hand,

$$ \sum _{k=0}^{n-1} \sup |\omega _k(\cdot , t)| \lesssim \sum _{k=0}^{n-1} A^{R^k} \lesssim A^{R^{n-1}} $$

For $k > n$, since $t \le T_{n+1} \le T_k$, $\omega _k(x, t) = 0$, so $\sup |\omega (\cdot , t)| \gtrsim A^{R^n}$. Then

$$\begin{aligned} \int _0^1 \sup |\nabla u(\cdot , t)|dt&\ge \sum _{n=0}^\infty \int _{2T_{n+1}-1}^{T_{n+1}} \sup |\omega (\cdot , t)|dt\\&\gtrsim \sum _{n=0}^\infty (1 - T_{n+1})A^{R^n} \gtrsim \sum _{n=0}^\infty R^{n+1}\ln (AN^s) = +\infty . \end{aligned}$$

## Data Availability

Data sharing is not applicable to this article as no new data were created or analysed in this study.

## Footnotes

1 By a rescaling argument, if the theorem holds for *some* $\nu >0$, then it holds for *all* $\nu >0$.

2 The right-hand side is not divergence free, so we need some $e_{n+3}$ component to fix that, which is small compared to the main term because of the oscillations in $x_{n+1}$ and $x_{n+3}$ have different frequencies.

3 It is well known that solutions of a generic linear recurrence *equation* are linear combinations of $R_j^n$, where $R_j$ is a root of the characteristic equation.

## References

[1] Barbato, D., Morandin, F., Romito, M.: Global regularity for a slightly supercritical hyper dissipative Navier–Stokes system. *Anal. PDE* **7**(8), 2009–2027, 2014

[2] Boutros, D.W., Gibbon, J.D.: Phase transitions in the fractional three dimensional Navier–Stokes equations. *Nonlinearity* **37**, 045010, 2024

[3] Caffarelli, L., Kohn, R., Nirenberg, L.: Partial regularity of suitable weak solutions of the Navier–Stokes equations. *Commun. Pure Appl. Math.* **35**(6), 771–831, 1982

[4] Chae, D.: On the regularity conditions for the Navier–Stokes and related equations. *Rev. Mat. Iberoam.* **23**, 371–384, 2007

[5] Chen, J.: Remarks on the smoothness of the asymptotically self-similar singularity in the 3D Euler and 2D Boussinesq equations. https://arxiv.org/pdf/2309.00150.pdf

[6] Chen, J., Hou, T.: Finite time blowup of 2D Boussinesq and 3D Euler equations with velocity and boundary. *Commun. Math. Phys.* **383**(3), 1559–1667, 2021

[7] Chen, J., Hou, T.: Stable nearly self-similar blowup of the 2D Boussinesq and 3D Euler equations with smooth data I. arXiv:2210.07191

[8] Chen, J., Hou, T.: Stable nearly self-similar blowup of the 2D Boussinesq and 3D Euler equations with smooth data II: Rigorous numerics. arXiv preprint: arXiv:2305.05660

[9] Cheskidov, A.: Blow-up in finite time for the dyadic model of the Navier–Stokes equations. *Trans. Am. Math. Soc.* **360**(10), 5101–5120, 2008

[10] Cheskidov, A., Dai, M., Friedlander, S.: Dyadic models for fluid equations: a survey. *J. Math. Fluid Mech.* **25**(3), 26, 2023

[11] Colombo, M., De Lellis, C., Massaccesi, A.: The generalized Caffarelli–Kohn–Nirenberg theorem for the hyperdissipative Navier–Stokes system. (2017). arXiv preprint arXiv:1712.07015

[12] Constantin, P., Fefferman, C.: Direction of vorticity and the problem of global regularity for the Navier–Stokes equations. *Indiana Univ. Math. J.* **42**(3), 775–789, 1993

[13] Cordoba, D., Martinez-Zoroa, L.: Blow-up for the incompressible 3D-Euler equations with uniform force. arXiv preprint arXiv:2309.08495.

[14] Cordoba, D., Martinez-Zoroa, L., Zheng, F.: Finite time singularities to the 3D incompressible Euler equations for solutions in . *Ann. PDE* **11**(2), 56, 2025

[15] De Rosa, L.: Infinitely many Leray–Hopf solutions for the fractional Navier–Stokes equations. *Commun. Partial Differ. Equ.* **44**(4), 335–365, 2019

[16] Escauriaza, L., Seregin, G.: Sverak: solutions of Navier–Stokes equations and backward uniqueness. *Uspekhi Mat. Nauk* **58**(2), 211–250, 2003

[17] Elgindi, T.M.: Finite-time singularity formation for solutions to the incompressible Euler equations on . *Ann. Math.* **194**(3), 647–727, 2021

[18] Elgindi, T.M., Ghoul, T.-E., Masmoudi, N.: On the Stability of Self-similar Blow-up for Solutions to the Incompressible Euler Equations on . *Camb. J. Math.* **9**(4), 1035–1075, 2021

[19] Elgindi, T.M., Jeong, I.-J.: Finite-time singularity formation for strong solutions to the axisymmetric 3D Euler equations. *Ann. PDE* **5**(2), 1–51, 2019

[20] Elgindi, T.M., Pasqualotto, F.: From instability to singularity formation in incompressible fluids. arXiv:2310.19780

[21] Fan, J., Fukumoto, Y., Zhou, Y.: Logarithmically improved regularity criteria for the generalized Navier–Stokes and related equations. *Kinet. Relat. Models* **6**, 545–556, 2013

[22] Fefferman, C.: Existence and smoothness of the Navier–Stokes equation. *Millennium Prize Probl.* **57**(67), 22, 2006

[23] Hölder, E.: Über die unbeschränkte Fortsetzbarkeit einer stetigen ebenen Bewegung in einer unbegrenzten inkompressiblen Flüssigkeit. *Math. Z.* **37**(1), 727–738, 1933

[24] Hou, T.: The potentially singular behavior of the 3D Navier–Stokes equations. *Found. Comput. Math.* **23**(6), 2251–2299, 2023

[25] Katz, N.H., Pavlović, N.: A cheap Caffarelli–Kohn–Nirenberg inequality for the Navier–Stokes equation with hyper-dissipation. *Geom. Funct. Anal.* **12**(2), 355–379, 2002

[26] Katz, N.H., Pavlović, N.: Finite time blow-up for a dyadic model of the Euler equations. *Trans. Am. Math. Soc.* **357**(2), 695–708, 2005

[27] Kim, J.-M.: Some regularity criteria for the 3D generalized Navier-Stokes equations. *Z. Angew. Math. Phys.* **72**(3), 9, 2021

[28] Kwon, H., Ożański, W.S.: Local regularity of weak solutions of the hypodissipative Navier–Stokes equations. *J. Funct. Anal.* **282**(7), 109370, 2022

[29] Leray, J.: Essai sur le mouvement d’un fluide visqueux emplissant l’espace. *Acta Math.* **63**, 193–248, 1934

[30] Lions, J.-L.: Quelques méthodes de résolution des problèmes aux limites non linéaires. Dunod, Gauthier-Villars, Paris (1969)

[31] Mercado, J.M., Guido, E.P., Sánchez-Sesma, E.P., Íñiguez, A.J., González, A.: Analysis of the Blasius’s formula and the navier–stokes fractional equation. In: Fluid Dynamics in Physics, Engineering and Environmental Applications, Environmental Science and Engineering, Springer, Berlin, Heidelberg, 2013, pp. 475–480.

[32] Miller, E.: Finite-time blowup for an Euler and hypodissipative Navier–Stokes model equation on a restricted constraint space. arXiv:2307.03434v3

[33] Nakai, K.: Direction of vorticity and a refined regularity criterion for the Navier–Stokes equations with fractional Laplacian. *J. Math. Fluid Mech.* **21**, 1–8, 2019

[34] Ożański, W.S.: Partial regularity of Leray–Hopf weak solutions to the incompressible Navier-Stokes equations with hyperdissipation. *Anal. PDE* **16**(3), 747–783, 2023

[35] Robinson, J., Rodrigo, J.L.: The Three-Dimensional Navier–Stokes Equations. Cambridge University Press, Cambridge (2016)

[36] Seregin, G., Sverak. V.: Navier-Stokes equations with lower bounds on the pressure. *Arch. Ration. Mech. Anal.* **163**, 65–86, 2002

[37] Tang, L., Yu, Y.: Partial regularity of suitable weak solutions to the fractional Navier–Stokes equations. *Commun. Math. Phys.* **334**(3), 1455–1482, 2015

[38] Tang, L., Yu, Y.: Erratum to: partial regularity of suitable weak solutions to the fractional Navier-Stokes equations. *Commun. Math. Phys.* **335**, 1057–1063, 2015

[39] Tao, T.: Global regularity for a logarithmically supercritical hyperdissipative Navier-Stokes equation. *Anal. PDE* **2**(3), 361–366, 2009

[40] Tao, T.: Finite time blowup for an averaged three-dimensional Navier–Stokes equation. *J. Am. Math. Soc.* **29**(3), 601–674, 2016

[41] Wu, J.: Lower bounds for an integral involving fractional Laplacians and the generalized Navier-Stokes equations in Besov spaces. *Commun. Math. Phys.* **263**, 803–831, 2006

[42] Wolibner, W.: Un theorème sur l’existence du mouvement plan d’un fluide parfait, homogène, incompressible, pendant un temps infiniment long. *Math. Z.* **37**(1), 698–726, 1933

[43] Zhang, X.: Stochastic Lagrangian particle approach to fractal Navier–Stokes equations. *Commun. Math. Phys.* **311**(1), 133–155, 2012

